# Climate Change and *Aedes* Vectors: 21st Century Projections for Dengue Transmission in Europe

**DOI:** 10.1016/j.ebiom.2016.03.046

**Published:** 2016-04-02

**Authors:** Jing Liu-Helmersson, Mikkel Quam, Annelies Wilder-Smith, Hans Stenlund, Kristie Ebi, Eduardo Massad, Joacim Rocklöv

**Affiliations:** a Department of Public Health and Clinical Medicine, Epidemiology and Global Health, Umeå University, Umeå, Sweden; b Department of Molecular Biology, Umeå University, Umeå, Sweden; c Lee Kong Chian School of Medicine, Nanyang Technological University, Singapore; d University of Washington, Seattle, Washington, USA; e School of Medicine, University of Sao Paulo, Brazil

**Keywords:** Dengue, Vectorial capacity, *Aedes aegypti*, *Aedes albopictus*, Temperature, Climate change

## Abstract

Warming temperatures may increase the geographic spread of vector-borne diseases into temperate areas. Although a tropical mosquito-borne viral disease, a dengue outbreak occurred in Madeira, Portugal, in 2012; the first in Europe since 1920s. This outbreak emphasizes the potential for dengue re-emergence in Europe given changing climates. We present estimates of dengue epidemic potential using vectorial capacity (*VC*) based on historic and projected temperature (1901–2099). *VC* indicates the vectors' ability to spread disease among humans. We calculated temperature-dependent *VC* for Europe, highlighting 10 European cities and three non-European reference cities. Compared with the tropics, Europe shows pronounced seasonality and geographical heterogeneity. Although low, *VC* during summer is currently sufficient for dengue outbreaks in Southern Europe to commence–if sufficient vector populations (either *Ae. aegypti* and *Ae. albopictus*) were active and virus were introduced. Under various climate change scenarios, the seasonal peak and time window for dengue epidemic potential increases during the 21st century. Our study maps dengue epidemic potential in Europe and identifies seasonal time windows when major cities are most conducive for dengue transmission from 1901 to 2099. Our findings illustrate, that besides vector control, mitigating greenhouse gas emissions crucially reduces the future epidemic potential of dengue in Europe.

## Highlights

• Past and present assessments of *VC* indicate strong seasonal patterns in temperate climates' dengue epidemic potential.
• Current *VC* intensity could permit summer dengue epidemics in Southern Europe driven by either *Aedes* vector, where present.
• Extent of spatial and temporal *VC* changes depend on vector characteristics and projected greenhouse gas emissions scenarios.
• With climate change, future *VC* intensifies: shifting northward and prolonging the season suitable for dengue epidemics.
• By the 21st century's end, seasonal dengue outbreaks could emerge in much more of Europe if *Aedes* vectors were established.
• Achieving the Paris Agreement's emission reduction commitments could decelerate the increasing threat of dengue to Europe.

Globalization and climate change can increase the geographic spread of vector-borne diseases. Among those, dengue, a mosquito-transmitted viral disease, causes up to 390 million human infections annually. This study evaluates potential for dengue outbreaks in Europe based on climate conditions. Estimated suitability (1901–2099) for dengue outbreaks is expanding presently from Southern Europe northward and lengthening seasonally up to eight months around the summer; however, the projected extent, intensity and duration depend partially on greenhouse gas emissions. We conclude that limiting emissions thereby mitigating climate change could substantially reduce the likelihood of dengue transmission events in Europe during the 21st century.

## 1 Introduction

Globalization and climate change may increase the risk of the geographic spread of vector-borne diseases (Hales et al., 2002, Liu-Helmersson et al., 2014, McMichael, 2013, Murray et al., 2013; World Health Organization (WHO), 2015). Changes in temperature variation have profound impacts on mosquito populations, and perhaps as important as changes in mean temperatures, if not more (Lambrechts et al., 2011, Vasseur et al., 2014). Since 1950, diurnal temperature extremes increased across the globe (Vasseur et al., 2014) and magnitudes of annual temperature cycles increased by 0.4 °C in temperate regions. This could result in elevated vulnerability within Europe for the introduction and re-establishment of vector-borne diseases such as dengue.

Dengue is a climate sensitive mosquito-borne viral disease that is generally found in the tropics and subtropics (Murray et al., 2013). According to the World Health Organization (World Health Organization (WHO), 2012) and recent assessment on global burden of diseases (GBD2013) (Murray et al., 2015), dengue is now the most important arboviral disease worldwide that has the largest increases among infectious diseases over the last 20 years. The recent estimates indicate as many as 390 million infections per year (Bhatt et al., 2013). *Aedes* mosquitoes are the vectors for the four dengue virus serotypes: DENV 1–4 (World Health Organization (WHO), 2012). *Ae. aegypti* is the primary vector associated with most major dengue epidemics, while *Ae. albopictus*, the secondary vector, is less efficient (Lambrechts et al., 2010).

Of major concern is the geographic expansion of dengue viruses and vectors to new areas (Lambrechts et al., 2010; World Health Organization (WHO), 2009). Reasons for the expansion are complex; however, main contributing factors include the introduction of *Aedes* mosquitoes by shipping (Reiter, 1998) and increasingly importation of the dengue virus via viremic travelers (Leder et al., 2013, Wilder-Smith and Gubler, 2008). Subsequent establishment of vectors after introduction can only be possible if suitable climate and ecological conditions exist. Having established populations of *Aedes* vectors and the conducive climatic conditions, in early autumn 2014, Tokyo, Japan recorded the first epidemic of dengue since the Second World War (Quam et al., 2016). Europe is also suitable for the establishment and re-establishment of *Aedes* mosquitoes as evidenced by the following: *Aedes aegypti* was historically present in many European countries including UK and France (1919), Spain (up to 1953), Portugal (up to 1956), and recently the Netherlands, Russia and Georgia (European Centre for Disease Prevention and Control (ECDC), 2014; European Centre for Disease Prevention and Control (ECDC), 2015). In Madeira, Portugal, *Ae. aegypti* was documented until 1977–79 and then was re-established in 2004 and 2005. Madeira experienced its first major dengue outbreak in 2012/2013, with more than 2000 cases (European Centre for Disease Prevention and Control, 2012, Wilder-Smith et al., 2014a). The rapidly expanding range of *Ae. albopictus* in Europe (Lambrechts et al., 2010; European Centre for Disease Prevention and Control (ECDC), 2013) resulted in the first known autochthonous dengue cases in southern France and Croatia in 2010 (La Ruche et al., 2010) in addition to an importation *Ae. albopictus* driven outbreak of chikungunya in 2007 in Italy (Rezza et al., 2007). There are concerns that *Ae. aegypti* could be introduced from Russia and Georgia (Abkhazia) to Western Europe via air or sea traffic, and to Eastern Europe via road and sea traffic, in addition to the vectors' projected establishment in Southern Europe (Rogers, 2015). Current surveillance indicates that *Aedes* vectors have been introduced or established in much of the Mediterranean coast and as far north as the Netherlands (European Centre for Disease Prevention and Control (ECDC), 2015). Therefore, it is important to assess the dengue epidemic potential (DEP) in Europe.

A few studies have projected dengue risk and epidemic potential for Europe. They have used either statistical models (Bouzid et al., 2014, Rogers et al., 2014) or vectorial capacity (Liu-Helmersson et al., 2014, Patz et al., 1998), a Mathematical model, however, with limited range either geographically, temporally or in terms of climate scenarios. In this study, we intend to estimate DEP for Europe using vectorial capacity with increased range of climate scenarios and temporal resolution. Although DEP depends on many factors, this study focuses on the effect of temperature – past, present, and future – on vectorial capacity of *Aedes* mosquitoes.

Vectorial capacity (*VC*) describes the threshold condition for a vector's ability to spread disease among humans (Patz et al., 1998, Massad and Coutinho, 2012), representing the average daily number of secondary cases generated by one primary case introduced into a susceptible population (Liu-Helmersson et al., 2014). It depends on six vector parameters (Patz et al., 1998), which are highly influenced by ambient temperature, both its mean value and diurnal temperature range (DTR) (Liu-Helmersson et al., 2014, Lambrechts et al., 2011, Carrington et al., 2013). *VC* has been used to model DEP globally for both *Aedes* vectors (Patz et al., 1998, Brady et al., 2014). Very few models incorporated DTR (Patz et al., 1998) and the temperature dependent transmission probabilities per bite to both humans and vectors (Lambrechts et al., 2011) when describing DEP (Liu-Helmersson et al., 2014) or vector competence. Including these factors would change the projected estimates of the impacts of climate on DEP, given strong temperature dependence of transmission probabilities per bite in humans and vectors and strong association of DTR with *VC* and vector competence (Liu-Helmersson et al., 2014, Lambrechts et al., 2011).

In this study, we modeled *VC* to project DEP in Europe given changes in climate. Throughout this study, we have included DTR in all of our *VC* calculations. We expanded our previous relative *VC* model to *VC*, by including temperature dependent dynamics in the female vector-to-human population ratio (Liu-Helmersson et al., 2014) for both *Ae. aegypti* and *Ae. albopictus* under four projected emission scenarios with higher temporally and spatially resolution over two centuries. We estimated DEP for local dengue transmission, in terms of seasonality, intensity and duration, for Europe and examined ten European metropolitan cities ranging from North to South for the period 1901–2099 (for more details see Table S3 in the Supplementary information).

## 2 Methods

Vectorial capacity (*VC*) was used to estimate dengue epidemic potential (DEP). As shown in Equation (Hales et al., 2002; Patz et al., 1998, Massad and Coutinho, 2012), *VC* depends on six vector parameters:

(1) $$VC=\frac{ma^{2}b_{h}b_{m}e^{-\mu_{m}n}}{\mu_{m}}.$$

The six vector parameters used were 1) the average vector biting rate (*a*), 2) the probability of vector to human transmission per bite (*b*_*h*_), 3) the probability of human to vector infection per bite (*b*_*m*_), 4) the duration of the extrinsic incubation period – EIP (*n*), 5) the vector mortality rate (*μ*_*m*_), and 6) the female vector-to-human population ratio (*m*). The time unit is one day. Each of the vector parameters depends on temperature (Liu-Helmersson et al., 2014). The temperature relationships for the first five vector parameters, 1)–5), were obtained from the peer-reviewed literature for *Ae. aegypti*; details are described elsewhere (Liu-Helmersson et al., 2014). For *Ae. albopictus*, only two vector parameters, 1) and 5), were found in the literature with temperature dependent relationships: the mortality rate (*μ*_*m*_) and the total biting rate (*a*), which was taken as an inverse of the duration of gonotrophic cycle (Delatte et al., 2009). The remaining three parameters, 2)–4), in the *VC* were assumed to have the same temperature relation as those for *Ae. aegypti* (Liu-Helmersson et al., 2014). For *Ae. Albopictus*, the human biting rate is assumed to be 0.88 of the total biting rate based on the human and dogs experiment performed by Delatte et al. (2010). The probability of transmission per bite to human is assumed to be 0.7 of that for *Ae. aegypti*, based partially on the literature review conducted by Lambrechts et al. (2010). Due to a lack of reliable data, the female vector-to-human population ratio, *m*, is assumed to depend on temperature the same way as the life expectancy or inverse of the mortality rate, as used in a previous study (Brady et al., 2014). The maximum value of *m* (*m*_max_) is assumed to be 1.5.

The threshold cut-off for DEP was defined as VC* = 0.2 (day^− 1^). Here we assume that an epidemic potential is realized when *VC* reaches a level such that one infected person will infect at least one more person after dengue is introduced into a naïve population during his/her five-day infectious period (Liu-Helmersson et al., 2014, Nishiura and Halstead, 2007) (Supplementary information, Section S4). Sensitivity analysis was performed for the effect of the range of the infectious period (4–10 days) (World Health Organization (WHO), 2012; Centers for Disease Control and Prevention (CDC), 2015; Chan and Johansson, 2012) on the dengue transmission windows (Supplementary information Section S5.3, Fig. S5A for *Ae. aegypti* and Fig. S5B for *Ae. albopictus*). This corresponds to a range of thresholds for DEP from 0.1 to 0.25 (day^− 1^). We have chosen the threshold value of 0.2 (day^− 1^), which is closer to the higher end of this range, to be conservative in our results presented.

To generate recent European season-stratified maps of *VC* (Jan. 1, 2006–Dec. 31, 2015), daily temperature observations (minimum, maximum, and mean) from the E-OBS 12.0 dataset were used for each location gridded at 0.25 × 0.25° (about 25 × 25 km at the equator) latitude and longitude (Haylock et al., 2008). This daily *VC* calculation included interpolating DTR based on daily observations, then aggregated over the decade by season (Winter: December–February; Spring: March–May; Summer: June–August; Autumn: September–November). The seasonal averaged *VC* for the recent decade were displayed as maps for Europe for each season for both vectors and compared to a recent survey of vector distribution (European Centre for Disease Prevention and Control (ECDC), 2015) for areas known to have *Aedes* activity according.

To show seasonality of *VC* over a year, decade averages of *VC* for each month was displayed as a function of the month in a year. For the recent decade, 13 cities were chosen to compare seasonality of DEP. Ten European cities were selected to represent most of the European continent with different temperature zones from the north — Stockholm (latitude = 59.3) to the south — Málaga (latitude = 36.7) within the continent and Madeira (latitude = 32.7) outside the continent. Madeira is an autonomous region of Portugal having a dengue outbreak in 2012, and for convenience in this paper, we will use the name of *city* for all the nine cities and Madeira region. Three reference cities from tropical and sub-tropical regions outside Europe were chosen for comparison. Colombo and Singapore are located in Asia close to the equator and display high dengue endemicity (Gubler, 2011, Gubler and Clark, 1995) despite political and financial investment in dengue control. By contrast Miami, located in North America with a sub-tropical climate with more similarity in environmental and social economic conditions to some of Southern Europe, has reported autochthonous dengue transmission occasionally, which typically does not develop into large scale epidemics (Theiler et al., 1960).

Diurnal temperature range is known to affect the competence of dengue vector *Aedes aegypti* (Lambrechts et al., 2011). Inclusion of DTR in modeling DEP for *Ae. aegypti* using relative *VC* (*rVC*) has shown a great difference comparing without DTR (Liu-Helmersson et al., 2014), especially in the cold to temperate climate zones in Northern hemisphere. This is because *rVC* (*VC*) depends on DTR strongly, both the peak intensity and the position. When DTR increases from 0 °C to 20 °C, the peak height of *rVC* reduced from 1.37 to 0.47 day^− 1^; the peak position of *VC* reduces from 29 °C to 20 °C. Therefore, in models including DTR, temperate climate zones with larger DTR will have greater DEP, while tropical areas with less DTR will have lesser DEP than estimated by models using mean temperature alone. This is particularly relevant to Europe, where DTR is greater than tropical areas.

From the Climate Research Unit (CRU) online database, time series (CRU-TS 3.22) of gridded (0.5 × 0.5 degrees) monthly averages of daily temperature observations (minimums, maximum, and mean) were obtained for Europe for the period January 1, 1901 to December 31, 2013 (Jones et al., n.d.). Given the importance of DTR to temperate European climate, in all the *VC* calculations, diurnal temperature range (DTR) was included. DTR was reconstructed using a representative daily temperature for each 30 min through a piece-wise sinusoidal function based on the monthly average of daily minimum, maximum, and mean observations for each location (same temperature for each day of the month in each 0.5 × 0.5 grid – See Supplementary information S3 for details). To illustrate the combined effect of DTR and mean temperature to *VC*, heat maps were generated for both *Aedes* vectors (Fig. S2 (d) & (d) in the Supplementary information).

To show the seasonal window and its change over time, a 30-year average was used for each monthly averaged *VC* at three periods – the beginning of the 20th century (1901–1930), at the turn from 20th to 21st century (1984–2013), and the end of the 21st century (2070–2099). Future *VC* was calculated using projected climate under four greenhouse gas emission pathways (RCP2.6, RCP4.5, RCP6.0 and RCP8.5) (Weyant et al., 2009) based on CMIP5 (Taylor et al., 2011, Warszawski et al., 2014) atmosphere-ocean general circulation models. For each emission pathway, CMIP5 temperature datasets (min, max, mean resolution 0.5 × 0.5°) were used (Taylor et al., 2011, Warszawski et al., 2014). The *VC* was calculated for each of the five global models (NorESM1-M, MIROC-ESM-CHEM, IPSL-CM5A-LR, HadGEM2-ES and GFDL-ESM2M) and then averaged over the five models (Taylor et al., 2011, Warszawski et al., 2014). We used these models as an ensemble to form a multi-model mean, with the intention of providing results that are based on greater consensus. The four RCP scenarios describe the possible range of radiative forcing of greenhouse gases in the year 2100 (+ 2.6, + 4.5, + 6.0, and + 8.5 W m^− 2^, respectively) (Weyant et al., 2009). *VC* calculations were aggregated by decade to show the trends in DEP over two centuries. A selection of the outputs of these projection-based *VC* calculations was also mapped for RCP2.6 and RCP8.5 for both species to show the changes in DEP across Europe under scenarios of greater or lesser emission mitigation.

To calculate the intensity and duration of dengue transmission, a seasonality curve was generated by plotting the decadally averaged *VC* as a function of month for each of the 10 cities first and then for each decade over the two centuries. The intensity of DEP was estimated by averaging the *VC* over the highest three consecutive months in the seasonality curve. The duration of transmission season was estimated by the intersections of the seasonality curve with the line defining the threshold condition (*VC* = 0.2 (day^− 1^)). The differences between the two intersections gave the number of months that *VC* was over the threshold. This was repeated for each decade over the two centuries and for each of the 10 European cities. Over the two centuries decadally averaged *VC* were from year zero to nine for each decade except the two decades with 9-year period: 1901–1909, 2011–2019 due to that both CRU and CMIP5 data started from year one in the data set: 1901 and 2011.

Sensitivity analyses were carried out and the results were included in the Supplementary Information, where section and figure numbers were marked with “S”. Using Monte Carlo simulations, 95% Credible Intervals (CI) (Pericchi and Walley, 1991) of *VC* were estimated for temperatures ranging from 10 °C to 32.5 °C. The variability in each of the six vector parameters was simulated assuming a normal random distribution. At each temperature under the random generation of parameters, *VC* was calculated based on Eq. (1). Repeating this process 1000 times for each temperature, the 2.5th and the 97.5th percentiles of the *VC* were estimated to give the values of *VC* ± 95% CI. Using the fitting functions (*VC* ± 95% CI vs. temperature) as the basic equations and temperature data as input, we estimated *VC* ± 95% CI for the ten cities over two centuries, including DTR (Section S5.1–5.2, Figs. S3–S4 and Table S2 in Supplementary information). In addition, sensitivity of *VC* to threshold values (VC*) and the maximum value of the female vector-to-human population ratio (*m*_max_) were also estimated based on Eq. (1). We compared three values: VC* = 0.1, 0.2, 0.25 day^− 1^ based on the infectious period of 4 to 10 days. The results were expressed as dengue transmission duration over the rest of this century for two RCPs (Fig. S5). We varied *m*_max_ in three values: 1, 1.5, and 2, which was chosen to reflect partially the range of pupae-to-human population ratio (0.3 to 60) for *Ae. aegypti* (Focks et al., 2000). The results were expressed as the seasonality curves (averaged *VC* vs. months) over the recent decade for selected 13 cities (Fig. S6), and transmission intensity and duration over the two centuries for two RCPs (Figs. S7–S8) in the Supplementary information.

This study is part of the DengueTools project funded by the European Union Seventh Framework Programme FP7/2007–2013 under grant agreement no. 282589. The funders had no role in this study design, data collection, analysis and interpretation, and decision to publish or preparation of the manuscript. There is no pharmaceutical company involved in this study in any form.

## 3 Results

### 3.1 Current Seasonality of Dengue Epidemic Potential in Europe and Tropical/Subtropical Cities

**Fig. 1:**
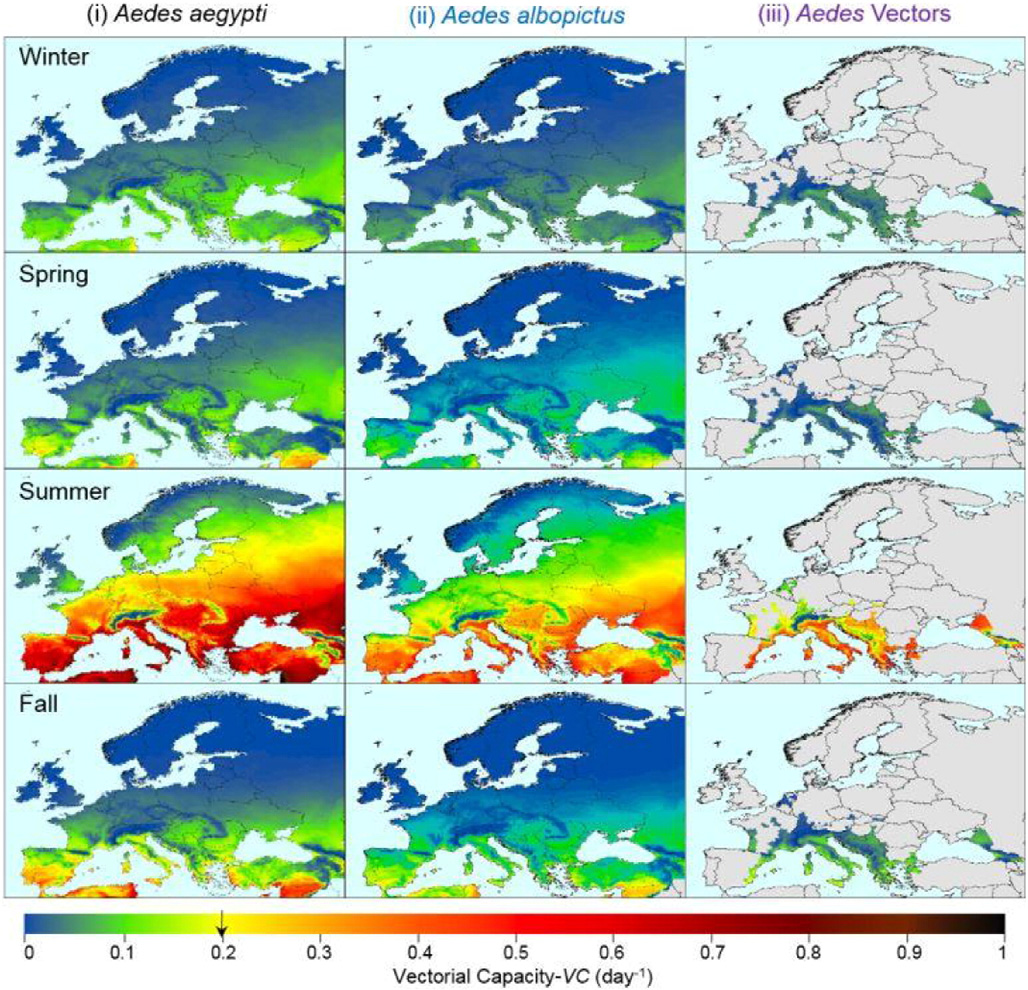
Season stratified maps of *VC* for Europe for *Ae. aegypti* (i), *Ae. albopictus* (ii), and in those areas having recently established and/or introduced *Aedes* vectors (iii) (European Centre for Disease Prevention and Control (ECDC), 2015; Wilder-Smith et al., 2014b). *VC* was calculated for each day of the period (Jan. 1, 2006–Dec. 30, 2015) and then seasonally aggregated over the decade. Winter: December–February; Spring: March–May; Summer: June–August; Autumn: September–November. DTR was included and *m*_max_ = 1.5. E-OBS 12.0 daily gridded (0.25 × 0.25°) temperature datasets were used (Haylock et al., 2008). The gray colored areas in this figure (iii) are those having unknown *Aedes* activity or for which survey information was unavailable (European Centre for Disease Prevention and Control (ECDC), 2015). The threshold value of 0.2 day^− 1^ is marked with an arrow on the yellow portion color bar.

Fig. 1 shows the season-stratified maps of Europe's DEP during the recent decade (2006–2015) for *Ae. aegypti* (Fig. 1(i)) compared to *Ae. albopictus* (Fig. 1(ii)) and current distribution of *Ae.* vectors either introduced or established (Fig. 1(iii)) (European Centre for Disease Prevention and Control (ECDC), 2015). Currently Europe is infested by *Ae. albopictus* mainly in the Mediterranean area but expanding northward, while only three areas have recently reported *Ae. aegypti*, Georgia and southwestern portions of Russia, in addition to Madeira Island, Portugal (not shown on map) (European Centre for Disease Prevention and Control (ECDC), 2015). The threshold value of 0.2 day^− 1^ corresponds to yellow color (see color bar). In areas with *VC* above this threshold (yellow-orange to dark red) during a given time period, dengue outbreaks may commence assuming prerequisite populations of vectors, susceptible human hosts, and virus introduction coincide. Areas during time periods displayed in blue to yellow green would not be expected to be suitable for dengue outbreaks to begin even if vectors were present and importations of dengue virus were persistent. Strong seasonality is apparent: *VC* was not sufficiently high in Europe in the winter, spring, and autumn seasons to allow dengue epidemic transmission to commence using the threshold value of 0.2 day^− 1^, except for small areas in the very southern parts of Southern Europe during spring and autumn. In the summer season, the majority of continental Europe for *Ae. aegypti* and southern and partially central parts of Europe for *Ae. albopictus* have climate conditions and corresponding *VC* that could sustain seasonal dengue epidemics. Therefore, if the primary vector, *Ae. aegypti*, established in the other parts of Europe in the future, it could have greater DEP than the established and invasive secondary vector, *Ae. albopictus*.

**Fig. 2:**
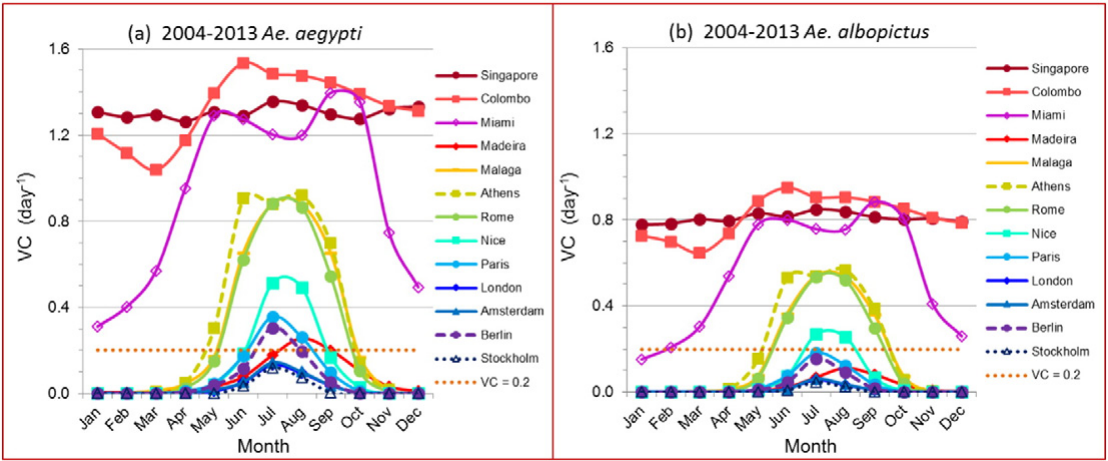
Seasonality of *VC* for 13 selected cities for *Ae. aegypti* (a) and *Ae. albopictus* (b). *VC* was averaged over the recent 10-year period (2004–2013) for each month of the year. DTR was included and *m*_max_ = 1.5 where *m* is the female vector to human population ratio. CRU-TS3.22 monthly gridded (0.5 × 0.5°) temperature data (Jones et al., n.d.) were used.

We compared 10 cities in Europe with three reference cities in tropical and sub-tropical regions. Fig. 2 shows *VC* averaged per month over the recent 10 years for *Ae. aegypti* (a) and *Ae. albopictus* (b). General decreases in *VC* were observed comparing the temperate European cities to the tropics, and the subtropics. Singapore and Colombo showed high and nearly constant year-round *VC*. Miami showed a broad peak from May to October, with *VC* values over the threshold (0.2 day^− 1^) year-round, indicating dengue epidemic transmission was theoretically possible. All European cities showed a strong and narrow seasonal transmission potential with overall lower *VC* values. Cities in Southern Europe exhibited higher and broader peaks in *VC* than the rest of Europe. *Ae. aegypti* showed higher *VC* than *Ae. albopictus*. Seven out of the 10 European cities were over the threshold for at least one month of the year for *Ae. aegypti*; only four cities were over the threshold for *Ae. albopictus*. For both vectors, no single city in Europe had sufficiently high *VC* to initiate endemic dengue transmission during the winter months. Therefore, during the past decade a dengue epidemic was possible only during the warmer months of the year in all three Southern European cities for both vectors and in some Central European cities for *Ae. aegypti* based on the CRU temperature data as input. Notice that this result based on monthly temperature is lower than the results shown in Fig. 1 using daily temperature as input – see limitation of this study in Discussion section and Section S1 in Supplementary information for more discussion. The rest of the results were based on monthly temperature.

### 3.2 Climate Change and the Dengue Epidemic Potential in Europe

**Fig. 3:**
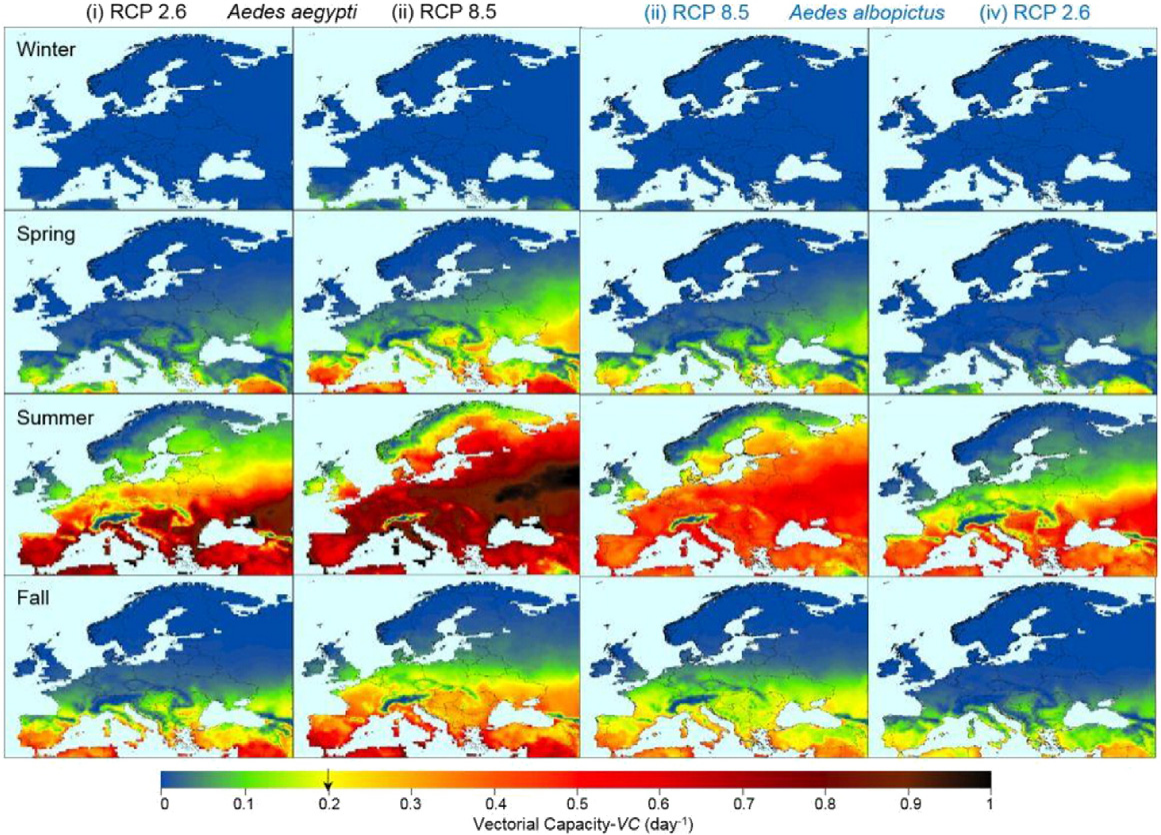
Season stratified maps of *VC* for Europe of the last decade of this century (2090–2099) under the greenhouse gas emission pathways RCP2.6 (i & iv) and RCP8.5 (ii & iii) for two *Aedes* vectors. The maps show the ten-year ensemble mean of five projection model-based *VC* calculations grouped by season (Taylor et al., 2011). Winter: December–February; Spring: March–May; Summer: June–August; Autumn: September–November. DTR was included and *m*_max_ = 1.5. Temperatures from five different global models (CMIP5 (Taylor et al., 2011, Warszawski et al., 2014)) were used as input for the projection and had original resolution of 0.5 × 0.5°. The threshold value of 0.2 day^− 1^ is marked with an arrow on the yellow portion of color bar.

Fig. 3 shows the season-stratified maps of Europe's DEP during the last decade of the 21st century under greenhouse gas emission pathways RCP2.6 (i & iv) and RCP8.5 (ii & iii) for two *Aedes* vectors. DTR was included and *m*_max_ = 1.5. Differences in *VC* for the four seasons are clearly shown. DEP is almost zero during the winter, then growing from small regions in the Southern Europe during the Spring, increasing intensity and expanding geographically during Summer, before contracting again in Fall. The differences in *VC* between the two climate scenarios and two *Aedes* vectors are apparent in Fig. 3. Under the most mitigation climate scenario (RCP2.6), during the Summer season, DEP is limited to the Southern and the Central Europe for both vectors. Under the business as usual climate scenario (RCP8.5), DEP extends into Northern Europe for both *Aedes* vectors. Under both climate scenarios, *Ae. aegypti* showed higher intensity in more areas than *Ae. albopictus*. By the end of this century, the highest DEP region during summer season would be in the Central Eastern parts of the Europe, in addition to parts of the coastal areas of Southern Europe already having high DEP in the recent decade as shown in Fig. 1.

**Fig. 4:**
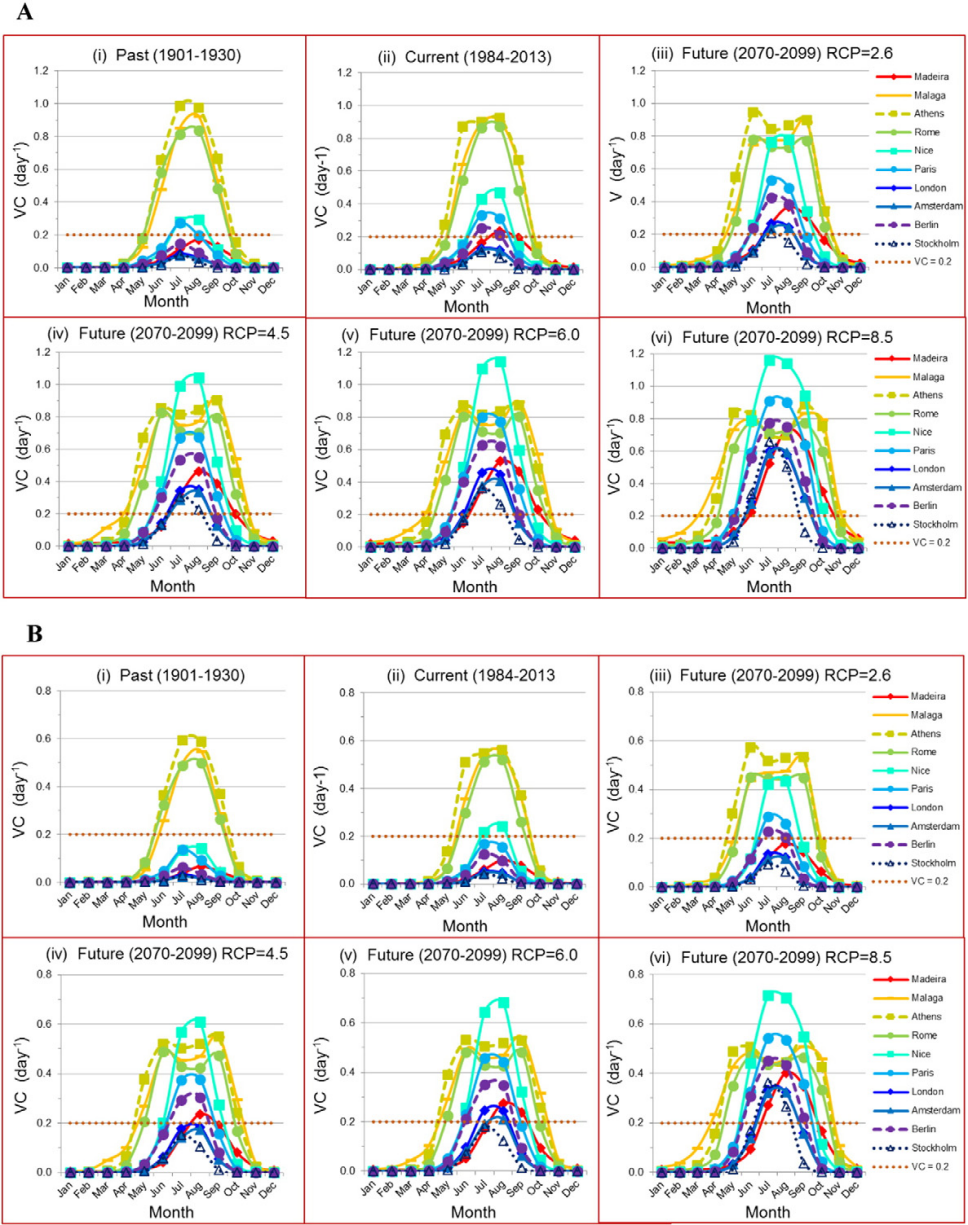
Seasonality comparison in *VC* among ten European cities over two centuries for *Ae. aegypti* (A) and *Ae. albopictus* (B). A 30-year averaged *VC* was plotted as a function of month for 3 different periods: Past (Fig. 3i), Current (Fig. 3ii), Future (Fig. 3iii–vi) under four different projected climate scenarios or emission pathways (RCP). DTR was included and *m*_max_ = 1.5. CRU-TS3.22 (Jones et al., n.d.) and CMIP5 (Taylor et al., 2011) gridded (0.5 × 0.5°) temperature data were used. For each emission scenario, *VC* was averaged over five different global models (CMIP5 (Taylor et al., 2011, Warszawski et al., 2014)).

Fig. 4 shows the trend in seasonality over two centuries (1901–2099) for the 10 European cities for *Ae. aegypti* (Fig. 4A) and for *Ae. albopictus* (Fig. 4B). For each city, 30-year averaged *VC* was estimated for three periods: Past (1901–1930) (i), Current (1984–2013) (ii), and Future (2070–2099) (iii–vi). We used historical temperature data from 1901 to 2013 and projected temperatures from 2011 to 2099 under five climate scenarios (CMIP5 (Taylor et al., 2011, Warszawski et al., 2014)) representing increasing emissions of greenhouse gases (Representative Concentration Pathways (RCPs) 2.6, 4.5, 6.0 and 8.5). Strong seasonality was observed in all cities over all periods and scenarios. Compared to the past, the current period shows an increase in the magnitude and the width of the peak in *VC* in central to Northern European cities (n = 7 including Madeira) while the Southern cities (n = 3) remained about the same (the magnitude of the peak was slightly reduced in Athens and increased in Rome).

The same trend was observed when comparing the future to the current period under different emission pathways. The higher the RCP, the higher the peak in *VC* for the Central to Northern seven cities including Madeira and the wider the window of transmission for all 10 cities. These observations hold for both *Aedes* vectors.

**Fig. 5:**
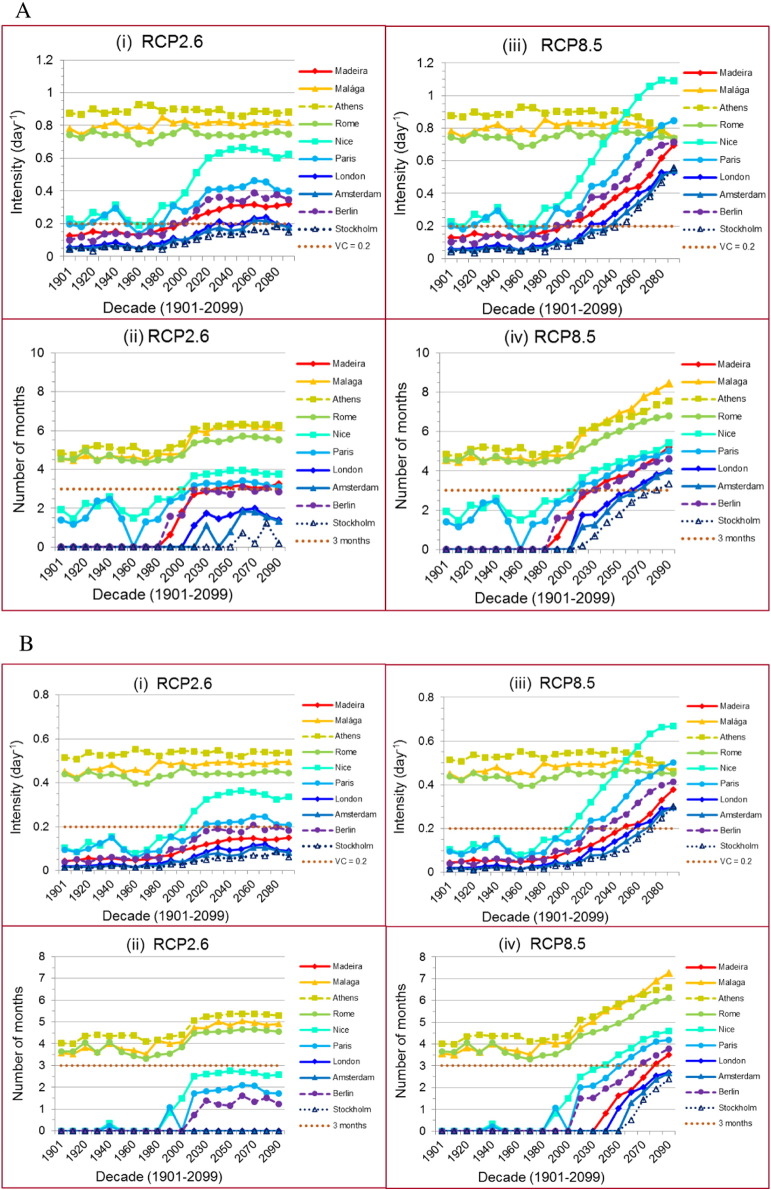
Transmission intensity and seasonal time window of dengue epidemic potential in 10 European cities for (A) *Ae. aegypti* (B) *Ae. albopictus*. Intensity was defined as the averaged *VC* over the highest consecutive 3-months for each decade. Transmission window was defined as the number of months when the decade averaged *VC* was over the threshold value (0.2 day^− 1^). Historical temperatures (CRU-TS3.22 (Jones et al., n.d.)) were used from 1901 to 2009. From 2011 to 2099, two emission pathways (CMIP5 (Taylor et al., 2011, Warszawski et al., 2014)) were evaluated: RCP2.6 (i & ii) and RCP8.5 (iii & iv).

Fig. 5 shows the intensity and duration of dengue transmission over two centuries for *Ae aegypti* (Fig. 5A) and *Ae. albopictus* (Fig. 5B). ‘Intensity’ was defined as the averaged *VC* over the highest three consecutive months for each decade, and ‘duration’ of transmission window was defined as the number of months when a decade's averaged *VC* was over the transmission threshold value (0.2 day^− 1^). Three months was used as the threshold in duration, reasoning that importation-driven epidemics of dengue would take several transmission generations to propagate in human and vector populations before the first reported cases of dengue are identified as was observed with Madeira in 2012 (Lourenço and Recker, 2014, Wilder-Smith et al., 2014b) and Japan in 2014 (Quam et al., 2016). Observed temperatures were used from 1901 to 2009. From 2011 to 2099, two emission pathways were evaluated: RCP2.6 (i–ii) and RCP8.5 (iii–iv).

In general, increasing trends in intensity and duration for DEP were observed in all cities. The intensity and duration markedly increased from 1970 to 2019 under both RCPs and for both vectors, except for the three Southern European cities: Malága, Athens and Rome, where intensity remained nearly constant due to decreasing sensitivity to slight temperature changes around the peak temperature for *VC*, as shown in Supplementary information (Section S4, Fig. S2(c)–(e)) and previously described for relative *VC* (Liu-Helmersson et al., 2014). From 2020 to the end of this century, this increase in intensity and duration is projected to level off under RCP2.6, while continuing to increase rapidly under RCP8.5 for both vectors, leading to very different projected trends.

During the current decade (2011–2019) under both RCPs, for *Ae. aegypti* the intensity threshold (0.2 day^− 1^) will be surpassed in seven cities (n_i_^a^ = 7) (Fig. 5A (i) & (iii)) and duration threshold (three months) in four cities (n_d_^a^ = 4) (Fig. 5A (ii) & (iv)). For *Ae. albopictus*, the intensity threshold will be surpassed for only four cities (n_i_^b^ = 4) in (Fig. 5B (i) & (iii)) and the duration threshold for three cities (n_d_^b^ = 3) (Fig. 5B (ii) & (iv)). From 2020 to 2099, under RCP2.6 for *Ae. aegypti*, the same results were observed for the intensity of (n_i_^a^ = 7) except two cities with short periods of over the threshold (London: 2050s–2070s and Amsterdam: 2060s–2070s); the number of cities with duration over the threshold will be increased from four to five at 2020s and to six starting 2040s to the end of this century (n_d_^a^ = 6). For *Ae. albopictus*, the number of cities with intensity over the threshold will be increased from four to five continuously (n_i_^b^ = 5) with one city (Berlin) over the threshold for only short time (2060s); the number of cities with duration over threshold remains the same (n_d_^b^ = 3). However, under RCP8.5, for *Ae. aegypti* all the 10 cities are projected to be over the thresholds in both intensity and duration (n_i_^a^ = 10 by 2050s, n_d_^a^ = 10 by 2080s); for *Ae. albopictus*, all 10 cities in intensity and seven cities in duration (n_i_^b^ = 10 by 2080s, n_d_^b^ = 7 by 2080s); this is a notable increase in DEP over the projections for RCP2.6 for both dengue vectors.

Assuming a duration of three months over the threshold in *VC* (dotted line in Fig. 5 (ii) & (iv)) is required for a dengue outbreak to occur (Liu-Helmersson et al., 2014), then in the past century (1901–2009) only Málaga, Athens and Rome had the potential for dengue outbreaks. However, for *Ae. aegypti*, in the current decade 2011–2019, Nice shows sufficient DEP under both RCPs. In the coming decades, under RCP2.6, there is little increase from the current to the future – only five cities (Málaga, Athens, Rome, Nice and Paris by 2020s) could have sufficient DEP. If RCP8.5 were to be realized, Paris would have sufficient DEP (2020s), followed by Madeira and Berlin (2030s), London (2060s), Amsterdam (2070s), and Stockholm (2080s). Therefore, by the end of this century, five cities under RCP2.6 and all 10 cities under RCP8.5 could have sufficient DEP – an *increase* of five cities from Central to Northern Europe (including Madeira) between the RCPs.

For *Ae. albopictus*, under RCP2.6 there is no difference between the future and past – only the Southern three cities, Málaga, Athens and Rome, had the potential for dengue outbreaks. Under RCP8.5, Nice will have sufficient DEP (2030s), followed by Paris (2060s), Berlin (2070s) and Madeira (2080s). Therefore, the gap in dengue epidemic duration between the two RCPs widens toward the end of this century - an *increase* of four cities between the two RCPs.

## 4 Discussion

Comparing to tropical and subtropical countries, Europe showed strong seasonality in DEP. No European city is projected to have year-round dengue epidemic transmission; the longest period would be eight months for *Ae. aegypti* and seven months for *Ae. albopictus* in Málaga by the end of the 21st century under RCP8.5.

As temperature increases with time from 1970s onward, Central (especially Nice) and Northern Europe has shown great increase in transmission intensity during summer while Southern Europe has shown decrease (Fig. 4). This is due to the combined effect of mean temperature and DTR as shown in Fig. S2 (c)–(f) in the Supplementary information.

Over time, the intensity and seasonal windows for DEP has increased and is projected to continue increasing. As a result, more cities will be over the *VC* threshold starting from the South and progressing to the North during this century. However, the rate of the increase depends on the emission pathway for both vectors, especially toward the middle of the century. The same trend is observed even when using the lower bound of *VC* (95% CI) for *Ae. aegypti* (see Supplementary information, Section S6.2). This implies a significant potential benefit, if policies for climate change mitigation are implemented such that future emissions more closely reflect RCP2.6.

Over the two centuries, we have observed that the DEP in Athens, Malága, and Rome are consistently over the threshold during part of the year. Nice stands out as having the most dramatic rise; by the 2060, Nice would surpass the intensity of the three Southern European cities; by the end of this century, during July–August Nice would be near the current summer peak intensity in Miami. Consistent with this, Nice was the site of the first reported autochthonous European cases in 2010 (La Ruche et al., 2010) and Athens had a massive dengue outbreak in 1927/28 (Theiler et al., 1960).

Since 1928, there has only been one dengue epidemic in Europe–Madeira 2012 with over 2000 cases transmitted through *Ae. aegypti* (European Centre for Disease Prevention and Control, 2012, Wilder-Smith et al., 2014b). Using local weather station temperature data for the current decade, *VC* for Madeira was well over the threshold from June to October (Fig. S1 in Supplementary information). Therefore, our findings are consistent with the large dengue outbreak that occurred in 2012 (European Centre for Disease Prevention and Control, 2012). The decline of new incident cases after November 9, 2012 was most likely due to declining *VC* because of cooler temperatures combined with enhanced vector control measures and public awareness. While our findings contribute valuable insight into the timing of the outbreak potential in Madeira, the introduction of vector predated the outbreak by years, and the climate based DEP predated both for a number of months each year. This illustrates that commencement of an actual dengue outbreak involves complex processes and more factors than what we have addressed here. Of note is that *VC* for Athens, Rome and Málaga is higher than that for Madeira even using local weather station data (UK Meteorological Office, n.d.), yet no dengue outbreaks recently occurred in those cities likely in part due to the absence of *Ae. aegypti*. However, *Ae. aegypti* may be introduced or re-introduced at any time. Our findings underpin the suitability of temperature dependent *VC* in countries such as Italy, Spain and Greece that could result in autochthonous dengue transmission should *Ae. aegypti* be imported and establish. Indeed, when *Ae. aegypti* was present in Greece in the early 20th century, a major dengue outbreak occurred in and around Athens in 1927–28 (Theiler et al., 1960). Furthermore, over 20 epidemics of yellow fever, another flavivirus (like dengue) transmitted via *Ae. aegypti*, occurred during the 18th and 19th centuries around British, Portuguese, and Spanish harbors, with the last outbreak being in Barcelona, Spain in 1821 (Morillon et al., 2002). With climate change, recent studies projected the re-establishment of *Ae. aegypti* in the coastal zones of Europe in 2080 (Rogers, 2015, Kraemer et al., 2015).

*Ae. albopictus* is already widely spread in much of Southern Europe, especially in the Mediterranean areas (Kraemer et al., 2015, Reiter, 2010). Comparing to *VC* of *Ae. aegypti* (Fig. 2(a)), *Ae albopictus* (Fig. 2(b)) had similar seasonal windows but with lower intensity such that only Southern European cities could currently have dengue epidemics. The main reason for the absence of dengue outbreaks in these cities could be one or all of the following factors: 1) Insufficient adult vector populations that are actively biting humans for an extended period to sustain a dengue outbreak (*Ae. albopictus* bites humans and animals (Lambrechts et al., 2010, Delatte et al., 2010)); 2) Insufficient infected humans with dengue for an extended period that could infect sufficient number of vectors to sustain a dengue outbreak (infected humans traveling back from dengue endemic areas are presumably isolated indoors or may have recovered before they traveled home); 3) *Ae. albopictus* is a less efficient vector for dengue transmission than what we estimated. We may have overestimated the *VC* for *Ae. albopictus* due to limitations in temperature dependent data and studies (Lambrechts et al., 2010).

However, *Ae. albopictus* was responsible for the epidemic transmission of chikungunya, an alphavirus, in Italy 2007 (Carrieri et al., 2011). Therefore, it is unlikely that at that time factor 1) - insufficient *Ae. albopictus* population - is the main reason for not having dengue outbreaks in Europe, unless *Ae. albopictus* more effectively drives transmission of chikungunya (Paupy et al., 2009) than dengue such that the current *Ae. albopictus* population are sufficient to drive chikungunya but too few to trigger dengue outbreaks (Dubrulle et al., 2009).

Each year, certain European travelers return home with dengue (i.e. 1207 cases reported in 2012 reported in EU/EEA, 884 of which lived in the EU countries currently having *Aedes* vectors (European Centre for Disease Prevention and Control (ECDC), 2014) and the number increased over time (Quam et al., 2015, Semenza et al., 2014, Tatem et al., 2006). If factor 2) is the main reason for preventing dengue outbreaks in Europe to date, this suggests outbreaks could occur anytime in the future when the infectious person/vector is in the suitable place at a suitable time, as was the case for chikungunya in Italy in 2007. If factor 3) is true, then the main concern for Europe is the potential introduction and establishment of *Ae. aegypti*, consistent with earlier studies (Liu-Helmersson et al., 2014, Lambrechts et al., 2010). Madeira like Miami highlights the difference between DEP and actually having dengue epidemic transmission. This can be multifactorial. The temporal and geographic range of DEP is likely to be spatially and temporally broader than the actual areas of transmission events (over estimations). On the other hand, Nice had local dengue transmission reported as early as September 2010 (La Ruche et al., 2010), which may indicate that the *VC* value was close to the threshold, and the Madeira and Japan outbreaks declined as *VC* went below the threshold conditions (Quam et al., 2016). When examining the DEP for Nice, our estimate of *VC* for *Ae. albopictus* was very close to the threshold as expected: July and August were over while September was under the threshold during the recent decade as shown in Fig. 2 (b). Therefore, we cannot ignore the dengue epidemic potential from *Ae. albopictus* in the Southern and Mediterranean areas of Europe as agreed by other studies (Lambrechts et al., 2010; European Centre for Disease Prevention and Control (ECDC), 2014; Bouzid et al., 2014, Rogers et al., 2014, Whitehorn et al., 2015, Benedict et al., 2007).

The time variable in this study was longitudinal – from 1901 to 2099 divided in 20 decades, in which for each decade monthly *VC* was estimated. All the existing models on dengue risk mapping regardless the model types – mathematical or statistical, give one or a few cross-sectional estimations, such as, one year (Rogers, 2015, Rogers et al., 2014, Patz et al., 1998) or a few decades' averaged snapshot (Bouzid et al., 2014, Patz et al., 1998, Brady et al., 2014, Louis et al., 2014, Naish et al., 2014). Although statistical models (Bouzid et al., 2014, Rogers et al., 2014) were used to estimated climate change impacts on dengue fever risk in Europe (more discussion in Supplementary information, Section S7.1), very limited information (Patz et al., 1998) can be found on seasonality in the current literature for European DEP or dengue risk over an extended time period under all climate change scenarios. To our knowledge, this is the only study on the European DEP communicating findings at the monthly time scale over two centuries including historical observations and four climate projections into the future. This information is important for dengue control planning and may in turn underscore the need for emissions reductions, echoing and supporting ratification of the Paris Agreement, a treaty on greenhouse gas emission reductions formed at the 2015 Paris Climate Conference (World Climate, 2015).

The limitations of this study are mainly the assumptions made and parameters/data used for estimating the DEP through *VC*. First, we assumed that the female vector-to-human population ratio (*m*) depends on temperature only. This is not exactly true in reality because vector populations change with the season due to climate including rainfall, vector ecology and control, etc. (Hii et al., 2012, Hales et al., 1996) The reason for making this assumption is that neither reliable data nor their temperature and rainfall dependent relationships are available. In addition, rainfall is known to affect the under-water stages of vector development (Yang, 2014, Tran et al., 2013). Once a vector population is established and abundant, temperature is the main climatic driver for dengue transmission and is included as the only climate variable in other mathematical modeling studies as well (Patz et al., 1998, Brady et al., 2014, Yang, 2014, Tran et al., 2013). To compensate for the assumption made in our model, we performed sensitivity analysis of *VC* to *m*_max_ – see Supplementary information (Section S6.4).

Second, the uncertainty in the vector and human parameters affect the value of DEP through *VC* and the threshold value for intensity. All six vector parameters depend on temperature. The female vector-to-human population ratio depends on temperature the same way as longevity (Brady et al., 2014). The rest five of them with relationships obtained from field and laboratory experimental studies (Liu-Helmersson et al., 2014). These relationships may vary depending on the environmental conditions of the study location/laboratory/design, vector and virus types. The uncertainty of each parameter and its temperature dependent relation were not available. Thus, a Monte Carlo simulation was used to estimate the uncertainty for each parameter and their effect on *VC*, from which the seasonality for the 10 European cities - see Supplementary information (Section S6.1–S6.2) for details. In addition, although we chose the threshold value more conservatively (0.2 day^− 1^ within the range of 0.1–0.25 day^− 1^ corresponding to infectious period of 4 to 10 days), the estimated intensity and duration for DEP should be viewed with caution. If different threshold values were used, the general trend and order of cities that would go over the threshold will hold, but the exact decade when the DEP goes over the threshold could change. However, using the threshold of 0.2 day^− 1^ in the analysis of both the outbreak in Madeira in 2012 and the 2014 dengue outbreak in Japan, we found that this threshold corresponded spatially and temporally with the novel transmission events (Quam et al., 2016). See Supplementary information (Section S6.3) for more discussion.

Third, for *Ae. albopictus*, only two parameters with temperature dependent relations were available in the literature (Delatte et al., 2009). The remaining parameters were assumed to have the same temperature dependent relationships as *Ae. aegypti*, although they were adjusted to the level of *Ae. albopictus* based on a literature review (Lambrechts et al., 2010). This would limit the accuracy of the estimated value of DEP for *Ae. albopictus*. See Supplementary information (Section S4) for more discussion.

Finally, the temperature data used from CRU and CMIP5 are monthly averages over gridded area of 0.5 × 0.5°. While the daily datasets from E-OBS for the maps in Fig. 1 have finer resolution (0.25 × 0.25°, daily), coarser resolutions (CRU and CMIP5) may underestimate DEP during the summer and overestimate during the winter for cities located along the costal lines. This accounts for the differences observed between Fig. 1 and Fig. 2 for major cities. Much of our analyses were based on outputs from the coarser temperature data sets (CRU and CMIP5). Therefore, the conclusions drawn are more conservative for the summer and overestimates for the winter (Fig. 5 intensity). See Supplementary Information (Sections S1 & S2) for more discussion.

## 5 Conclusion

We identified past, present, and future high-risk cities and time periods for potential dengue transmission in Europe based on temperature and daily temperature variation. Compared to countries where dengue is endemic, Europe showed strong seasonality in dengue epidemic potential (DEP) without possibility of year-round epidemic transmission. Compared over two centuries, we found a slow increase in intensity and duration of dengue transmission over the past century and more rapidly changing trajectories projected in the 21st century with the rate of change depending on the level of greenhouse gas emissions. Although Europe currently does not have a sufficiently high DEP year round, increasing periods with higher temperatures and greater temperature variation in the future due to climate change could elevate DEP along a south to north gradient. By the end of this century, DEP for *Ae. aegypti*, could expand to Northern Europe (all 10 cities studied) and up to eight months in Southern Europe under the highest emission pathway (RCP 8.5). Under the lowest emission pathway (RCP 2.6), it could expand to Nice and Paris for *Ae. aegypti* from the current three Southern European cities. For *Ae. albopictus* DEP could expand to all of the Central Europe (7 cities) under RCP 8.5; however, it would remain nearly as it is now under RCP 2.6 (three Southern Europe cities). Therefore, climate change mitigation (or lack thereof) could have a large impact on the seasonal window and geographic range for dengue transmission potential in Europe. Under the higher emission scenarios, increasingly larger parts of Europe would have the potential for autochthonous dengue transmission should *Ae. aegypti* be introduced and established. Such concerns were substantiated by the dengue outbreak in Madeira in 2012. The same concern extends to *Ae. albopictus* if higher greenhouse gas emissions than RCP2.6 would be realized.

Increasing globalization in travel and trade will intensify the importation of dengue viruses and the potential for further introduction of *Ae. aegypti* (Gubler and Clark, 1995). If such introductions coincide with suitable vectorial capacity, those cities at the time intervals identified in this study have potential for local dengue transmission. Dengue epidemic outbreak is a complex process involving many factors, with mean temperature and diurnal temperature range just being two of many. This study based on vectorial capacity helps to identify cities and areas with high DEP and the seasonal time windows when such cities are at the highest risk currently and in the future under climate change. Our findings illustrate that besides vector control, reducing greenhouse gas emissions is very important in reducing DEP for Europe especially toward the latter half of this century.

## Author contributions

JLH carried out the modeling, generated the calculations, and drafted the manuscript. MQ and HS obtained temperature datasets, created the maps, developed program code and assisted with the calculations and drafting of the manuscript. JR conceived the research, assisted with modeling and drafting of the manuscript. AWS contributed to the design and writing of the manuscript. EM assisted with the sensitivity analyses. KE provided scientific input into the manuscript and all authors discussed the results and contributed to the revision of the final manuscript.

## Competing financial interest statement

The authors declare that they have no competing financial interests.

## Appendix A Supplementary information

Supplementary material. Image 1
