# Supplementary material for: Climate Change and Aedes Vectors: 21st Century Projections for Dengue Transmission in Europe
Source: eBioMedicine. 2016 Apr 2;7:267–77. doi: 10.1016/j.ebiom.2016.03.046 (PMC4909611; doi:10.1016/j.ebiom.2016.03.046)
Supplement: Supplementary file 1 — Supplementary material. [file mmc1.pdf]

Article Highlights:

Climate Change and *Aedes* Vectors: 21st Century Projections for Dengue Transmission in Europe

- This study shows potential for future dengue outbreaks in Europe based on temperature-dependent vectorial capacity (*VC*)
- Past and present assessments of *VC* indicate strong seasonal patterns in temperate climates' dengue epidemic potential
- Current *VC* intensity could permit summer dengue epidemics in Southern Europe driven by either *Aedes* vector, where present
- Extent of spatial and temporal *VC* changes depend on vector characteristics and projected greenhouse gas emissions scenarios
- With climate change, future *VC* intensifies: shifting northward and prolonging the season suitable for dengue epidemics
- By the 21st century's end, seasonal dengue outbreaks could emerge in much more of Europe if *Aedes* vectors were established
- Achieving the Paris Agreement's emission reduction commitments could decelerate the increasing threat of dengue to Europe

Supplementary Information to Research Paper:

Climate Change and *Aedes* Vectors: 21st Century Projections for Dengue Transmission in Europe

Jing Liu-Helmersson[a*], Mikkel Quam[b], Annelies Wilder-Smith[a,c], Hans Stenlund[b], Kristie Ebi[a,d], Eduardo Massad[e], Joacim Rocklöv[a]

*Corresponding Author Email: jing.helmersson@umu.se

[a]Department of Public Health and Clinical Medicine, Epidemiology and Global Health, Umeå University, Umeå, Sweden;
[b]Department of Molecular Biology, Umeå University, Umeå, Sweden;
[c]Lee Kong Chian School of Medicine, Nanyang Technological University, Singapore;
[d]University of Washington, Seattle, Washington, USA.
[e]School of Medicine, University of Sao Paulo, Brazil.

A R T I C L E I N F O

Article History:
Received: 11 December 2015
Revised: 14 March 2016
Accepted: 30 March 2016
Published: 27 May 2016 (e-Pub: 2 April)

Key words:
Dengue
Vectorial Capacity
Aedes aegyptus
Aedes albopictus
Temperature
Climate Change

A B S T R A C T

Warming temperatures may increase the geographic spread of vector-borne diseases into temperate areas. Although a tropical mosquito-borne viral disease, a dengue outbreak occurred in Madeira, Portugal, in 2012; the first in Europe since 1920s. This outbreak emphasizes the potential for dengue re-emergence in Europe given changing climates. We present estimates of dengue epidemic potential using vectorial capacity (*VC*) based on historic and projected temperature (1901 - 2099). *VC* indicates the vectors' ability to spread disease among humans. We calculated temperature-dependent *VC* for Europe, highlighting 10 European cities and three non-European reference cities. Compared with the tropics, Europe shows pronounced seasonality and geographical heterogeneity. Although low, *VC* during summer is currently sufficient for dengue outbreaks in Southern Europe to commence–if sufficient vector populations (either *Ae. aegypti* and *Ae. albopictus*) were active and virus were introduced. Under various climate change scenarios, the seasonal peak and time window for dengue epidemic potential increases during the 21st century. Our study maps dengue epidemic potential in Europe and identifies seasonal time windows when major cities are most conducive for dengue transmission from 1901-2099. Our findings illustrate, that besides vector control, mitigating greenhouse gas emissions crucially reduces the future epidemic potential of dengue in Europe.

Research in context:

Globalization and climate change can increase the geographic spread of vector-borne diseases. Among those, dengue, a mosquito-transmitted viral disease, causes up to 390 million human infections annually. This study evaluates potential for dengue outbreaks in Europe based on climate conditions. Estimated suitability (1901-2099) for dengue outbreaks is expanding presently from Southern Europe northward and lengthening seasonally up to eight months around the summer; however, the projected extent, intensity and duration depend partially on greenhouse gas emissions. We conclude that limiting emissions thereby mitigating climate change could substantially reduce the likelihood of dengue transmission events in Europe during the 21st century

Content of Supplementary Information

S1. Comparison of temperature data - gridded CRU vs. local weather stations MIDAS
S2. Madeira: the comparison from different temperature data sources on *VC* estimation
S3. Temperature dependent vectorial capacity and female vector-to-human population ratio (*m*)
S4. Temperature dependent vector parameters for *Aedes albopictus* and vectorial capacity including DTR
S5. Vectorial Capacity (*VC*) and epidemic outbreak threshold
S6. Sensitivity Analysis of *VC* for *Aedes* vectors
  S6.1 Uncertainty of six vector parameters to *VC*: Monte Carlo simulation and *VC* ± 95%CI
  S6.2 *VC* ± 95%CI for ten European cities over three periods of the 21st century
  S6.3 Uncertainty of threshold (infectious period) to dengue transmission duration
  S6.4 Uncertainty of vector population (m_{max}) to transmission intensity and duration
S7. Comparison of this study with other statistical models and our previous work
  S7.1. Mathematical model vs. Statistical model, comparing with Bouzid et al's dengue risk mapping in Europe
  S7.2. Comparing with our previous study using relative *VC* for dengue epidemic potential

S1. Comparison of temperature data: gridded CRU vs. local weather station MIDAS data

Our study of dengue epidemic potential based on vectorial capacity (*VC*) uses temperature data as the input. For most of our results (Figures 2-4, S1, S4-S8), we used Climatic Research Unit (CRU) Time Series temperature data (CRU-TS3.22[1]) because of its consistent historic time span for over 100 years, 1901.1- 2013.12. The CRU data set is gridded to 0.5 x 0.5 degree resolution (about 50x50 km at the equator), based on analysis of over 4000 individual weather station records. This means that temperature values could be sensitive to their surrounding environment, such as oceans, through the spatial interpolation algorithm applied. Therefore, the consistency of temperatures were checked between data from CRU and local weather stations in the Met Office Integrated Data Archive System (MIDAS)[2].

Temperature data from CRU were monthly averaged means and the data from MIDAS were recorded every 3-hours (8 times daily). Therefore, MIDAS data were averaged for each month before comparing with CRU-TS3.10[3] data. Differences between the two data sets were calculated for each month from 1/2000 to 12/2009. The means and the standard deviations (SD) were calculated over the 10-year periods (Table S1).

As shown in Table S1, the two data sets are more or less identical except that deviations occur for cities having a large portion of coastal lines. An example is the coastal city of Funchal on the Portuguese island of Madeira, which in total is only about 740 km^2, less than one-third of a single grid in the CRU datasets. The comparison indicates that our results based on CRU data appear generally valid. However, MIDAS data may further improve the understanding of dengue *VC*, particularly in coastal cities, for time resolutions of less than a month, and for modelling diurnal variation more accurately.

The differences observed in the two datasets (CRU vs. MIDAS) were largest in Madeira (3.34 °C), then Nice (2.17 °C) and Malága (1.79 °C) among the European cities studied. T the *VC* values from CRU data for all the coastal cities may be underestimated during the summer and overestimated during the winter compared to its observed value from local weather station. The relatively small standard deviations (SD) observed for the comparison show a rather consistent difference between the data sources over time for each location.

Table S1. Differences in temperature between MIDAS[2] (local weather station) and the CRU-TS3.10[3] (gridded) data set. Temperatures from each source were averaged for the period 1/1/2000 – 12/31/2009.

City	T_{MIDAS}-T_{CRU} (SD) °C
Stockholm	0.56 (0.5)
Berlin	0.26 (0.3)
Amsterdam	0.17 (0.2)
Paris	-0.66 (0.4)
Nice	2.17 (0.2)
Rome	-0.62 (0.5)
Athens	1.34 (0.3)
Málaga	1.79 (0.7)
Madeira	3.34 (0.3)
Miami	-0.31 (0.1)
Colombo	0.94 (0.8)
Singapore	0.09 (0.2)

S2. Madeira: the comparison from different temperature data sources on *VC* estimation

We chose Funchal, Madeira to illustrate the difference in *VC* for the coastal cities. Figure S1 shows the average *VC* over the period 2004-2013 as a function of month, including DTR. In Figure S1(a), Madeira's temperature was calibrated to the local weather station MIDAS[4] data by adding 3.34 °C (see Table S1); temperature was not adjusted in Figure S1(b). Compared to using unadjusted CRU data, the peak height for Madeira increased and the seasonal window in *VC* over the threshold value increased from one month to five months when using adjusted CRU data. Therefore, using the original CRU data, our estimation of *VC* in Madeira was considerably lower than the values estimated from local weather station due to the lower averaged

temperature values from the climate data source. We assume that this bias continues to the future given that the grid of the future projection data corresponds to the CRU data. However, the overall trends over 200 years are not greatly affected by the CRU temperature data bias, although the exact decade that the DEP will be over the threshold may vary.

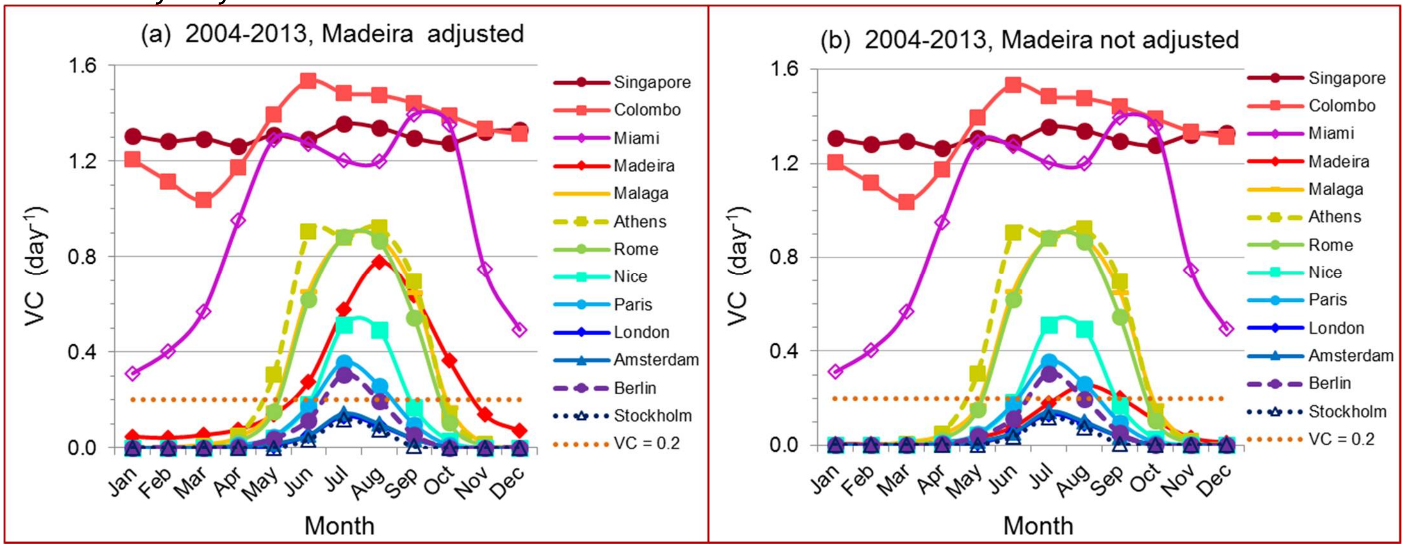

Figure S1. Seasonality of *VC* for 13 selected cities – ten European cities plus three reference cities from tropical and subtropical cities. *VC* was averaged over the period 2004-2013 for each month of the year, including DTR. CRU-TS3.22[1] monthly temperature data were used_ENREF_2 for all cities (Fig. S1(b) except Madeira where temperature was adjusted to MIDAS[2] (local weather station in Funchal) data (Fig. S1(a)).

S3. Temperature dependent vectorial capacity and female vector-to-human population ratio

As described in the Method section of the main article, vectorial capacity is defined as[4,5]

$$VC = \frac{ma^2 b_h b_m e^{-\mu_m n}}{\mu_m} \quad . \quad \text{(S1)}$$

The six vector parameters involved are 1) the average vector biting rate (a), 2) the probability of vector to human transmission per bite (b_h), 3) the probability of human to vector infection per bite (b_m), 4) the duration of the extrinsic incubation period (*n*), 5) the vector mortality rate (μ_m), and 6) the female vector-to-human population ratio (*m*). Each of the six vector parameters depends on temperature. The relationship between *VC* and temperature depends on the temperature relationship of each of the six individual vector parameters in equation (S1). The temperature relationships for the rest five vector parameters are found in literature for *Ae. aegypti* and described in detail elsewhere[6].

The temperature relationships of vector parameters provided the basis for incorporating the influence of DTR in *VC* (equation (1)). We created a new daily temperature profile (48 points) using the daily mean temperature (*T*) and the daily DTR by assuming a sinusoidal ½-hourly temperature variation between the two extremes (*T* ± DTR/2) within a period of 24 hours. The corresponding *VC* was calculated using this new daily temperature for each 30 min. of the day and then averaged over a day. When using monthly temperature data (maximum, mean and minimum) as input, we have assumed the same temperature for each day of the month. In the same way as with daily temperature (*T,* DTR), *T* is the mean temperature and DTR is between the maximum and mean, and between mean and the minimum temperature. DTR was incorporated in the calculated *VC* for each day (every 30 min. first and then averaged over the day). This daily *VC* is the same averaged *VC* over the month.

The female vector-to-human population ratio, *m*, is assumed to depend on temperature the same way as the life expectancy or inverse of the mortality rate. The longer the female mosquito lives, the higher the female population would be. The same reasoning has been used before[7]. A constant, *c*, with unit of 1/time has to be multiplied in order to keep the *m* in the right unit as shown in Eq. (S2) below.

$$m = \frac{c}{\mu_m} \quad \text{(S2)}$$

Here c is chosen such that the maximum value of m (m_{max}) is 1.5. Sensitivity analysis of *VC* to other values of m_{max} is discussed in Section S6.

S4. Temperature dependent vectorial capacity and vector parameters for *Aedes albopictus*

Aedes albopictus is an important dengue vector for Europe. Although being a secondary dengue vector, the widespread distribution of *Aedes albopictus* in Southern (Mediterranean) Europe warrants special attention[8-11]. However, the literature base is limited on temperature relationships.

Among the six vector parameters involved in the *VC* calculation[6,7] only the temperature dependence of mortality rate (μ_m) and human biting rate (a) were found[13]. On the other hand, both biting rate and mortality rate affect the *VC* most due to their quadratic (a) and exponential relation (μ_m) as shown in Equation (S1). The remaining parameters in the *VC* were assumed to have the same temperature relation as those for *Aedes aegypti*[76] with some justifications as discussed in the Method section of the main manuacript.

Figure S2 shows the temperature dependent relations of these two vector parameters and vectorial capacity for *Ae. albopictus* and using *Ae. aegypti* for comparison. As we know, *Ae. albopictus* bites both animals and humans[11,12]. Based on the human and dogs experiment[13], the values for biting rate (a) to human was taken as 0.88 of the total biting rate (a_T), which is taken as inverse of the duration of gonotrophic cycle[14]. The biting rate shown in Fig. S2(b) is for biting rate to human (a) after multiplying 0.88 to the total biting rate. At both low and high temperatures (less than 20 and higher than 31°C), *Ae. albopictus* has higher survival rate or the lower mortality rate as compared to *Ae. aegypti*, which will increase the *VC*. On the other hand, at these two ends of temperature (less than 20 and higher than 31°C), *Ae. albopictus* has lower biting rate than *Ae. aegypti* which will decrease the *VC*. Only in a very narrow tropical climate (between 25 and 31°C), *Ae. albopictus* could have higher *VC* than *Aedes aegypti* if the other vector parameters were the same for both vectors. For the rest of the three vector parameters used in the *VC* calculation, no temperature dependent relations were found in the literature. We used the same relations for *Aedes aegypti*[6]. However, a reduction of 0.7 was used for the probability of transmission per bite to human (b_h) for *Aedes albopictus* relative to that for *Ae. Aegypti*, based on laboratory experiments on relative oral susceptibility to DENV of the two *Aedes* vectors[14]. In these experiments, Lambretchts et al. found that relative to *Aedes aegypti*, the rate of midgut infection (b_m) for *Aedes albopictus* was increased by 0.08 and the rate of virus dissemination from the midgut (b_h) was reduced by 0.29 for mosquito colonization of less than 5 generations (Table 1)[12]. Since the change of b_m is small, we assume that was the same for the two vectors. For b_h, we converted the difference between the two vectors to ratios (0.5 - 0.7) at different temperatures (20 - 30 °C) based on the temperature dependent relation of b_h[15]. The highest ratio 0.7 was used to calibrate the probability of transmission per bite to human (b_h) for *Aedes albopictus* relative to that for *Ae. Aegypti* in the temperature dependent relation. Fig. S2(c) shows the overall effect of all parameters to *VC* for *Ae. albopictus* which is lower than that for *Ae. Aegypti* as expected.

Figure S2(c) and (d) show the overall effect of all parameters to the vectorial capacity for both vectors as a function of temperature. *VC* for *Ae. albopictus* is lower than that for *Ae. Aegypti* as expected. In these figures, we have also included the same relationships for five different DTRs. As we have found earlier with relative *VC*, *VC* depends on DTR strongly, both the peak intensity and the position. When DTR increases from 0°C to 20°C, the peak height of *VC* reduced from 2.06 to 0.66 day^{-1} for *Ae. aegypti* and from 1.25 to 0.39 day^{-1} for *Ae. Albopictus*; the peak position of *VC* reduces from 29°C for *Ae. aegypti* (29.3°C for *Ae. albopictus*) to 20°C for both *Aedes* vectors.

The combined effects of mean temperature and DTR on *VC* are more complicated. As shown in Fig. S2(e) for *Ae. aegypti* and Fig. S2(f) for *Ae. Albopictus*, *VC* as heat maps illustrate a non-linear relationships to temperature. If we take a horizontal cut of the map, as DTR increases, *VC* vs. mean temperature relation changes from a curve with a single peak to that with two or more peaks of different heights. If we take a vertical cut of the map at the peak temperature of 29°C, as DTR increases *VC* reduces monotonically; however, if the mean temperature is at 15°C, as DTR increases *VC* increases first and reaches a plateau, and eventually will decrease at very high DTR. Therefore, in models including DTR, temperate climate zones with larger DTR will have greater DEP, while tropical areas with less DTR will have lesser DEP than the estimate in models using mean temperature alone. This is particularly relevant to Europe, where DTR is greater than tropical areas.

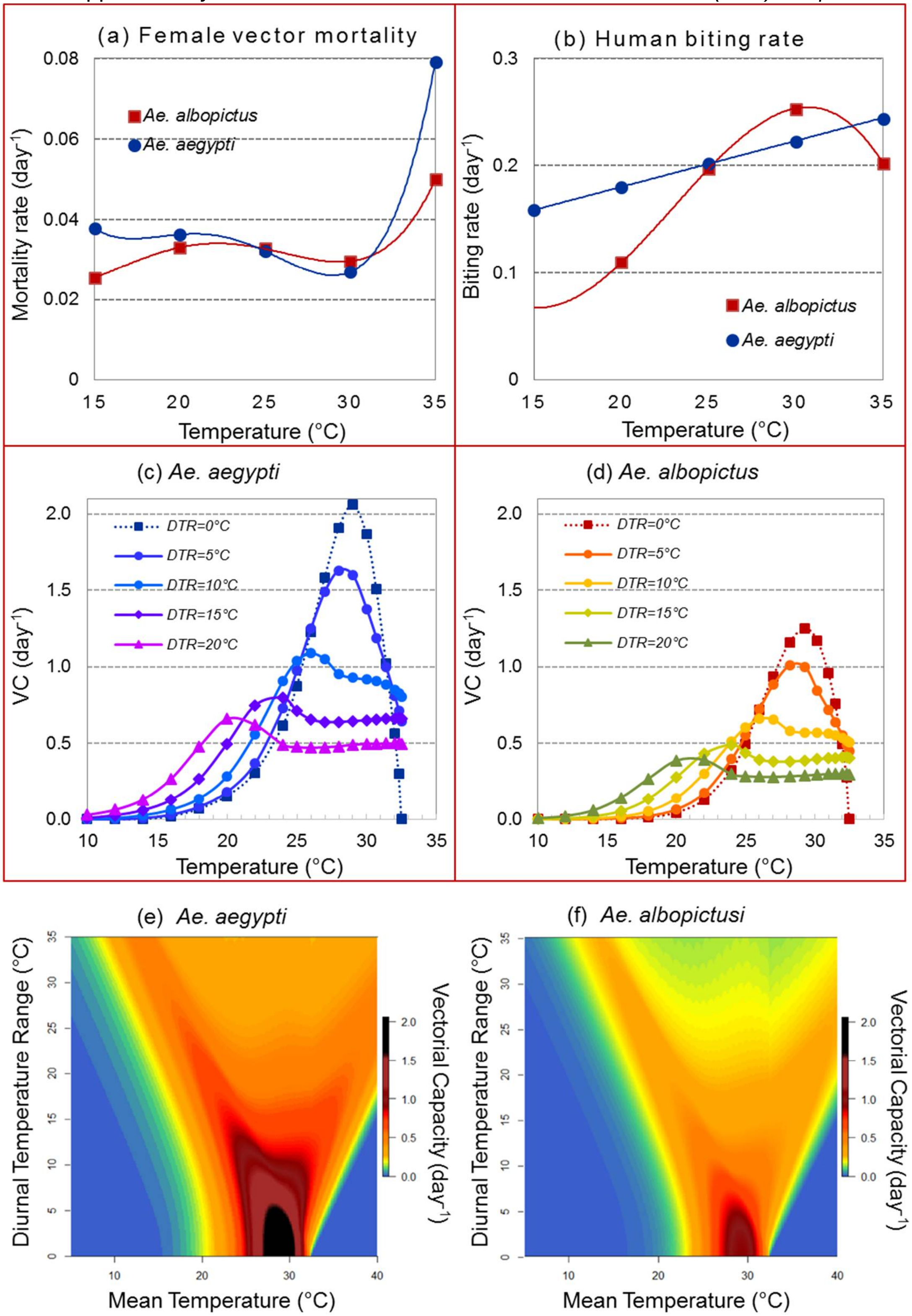

Figure S2. Temperature dependent vector parameters and vectorial capacity comparison for two dengue vectors - *Ae. aegypti*[16,17] and *Ae. Albopictus*[14]. Vector parameters are shown in Fig. S2(a) - Female vector mortality rate, and Fig. S2(b) - biting rate to human. The biting rate to human is assumed to be 0.88 of the total biting rate based on the human and dogs experiment[13]. The probability of transmission per bite to human (b_h) is assumed 0.7 of that for *Ae. Aegypti*[12]. Lines in Fig. S2(a) & (b) are fitting to 3rd (biting rate) to 4th (mortality rate) polynomial functions. Vectorial capacity and its dependence on temperature and DTR are shown in Fig. S2(c-f). The combined effects of mean temperature and DTR on *VC* are shown as heat maps for *Ae. aegypti* (Fig. S2(e)) and for *Ae. Albopictus* (Fig. S2(f)).

This combined effect of mean temperature and DTR helps us to understand the result in Fig. 4 in the main article. As temperature increases with time from 1970s onward, Central (especially Nice) and Northern Europe has shown great increase in transmission intensity during summer while Southern Europe has shown decrease. This is due to both the increase of mean temperature and differences in DTR. The larger DTR in Central to Northern Europe than the Southern Europe shifted the peak temperature of *VC* from 29°C for *Ae. aegypti* (29.3°C for *Ae. albopictus*) when DTR is 0°C to lower temperature. Together with increased mean temperature, *VC* for this region would be closer to the new peak value while the Southern Europe would move further away from the peak position with reduced *VC* value as shown in Fig. S2(c) – (f).

S5Vectorial Capacity (*VC*) and epidemic outbreak threshold

The basic reproduction number R_0 is usually used to describe the epidemic outbreak threshold. It represents the number of new cases generated by one infected person during his/her infectious period (T_h) when introduced into a totally susceptible population. Vectorial capacity represents the daily basic reproduction number[4-6] if using days as the unit for infectious period. An outbreak of an infectious disease occurs when R_0 is larger than 1[18,19]. *VC* is related to R_0 according to the expression:

$$VC = \frac{R_0}{T_h} \quad . \qquad \text{(S3)}$$

Where T_h is the human infectious period. The critical or threshold values (labeled as *) for an epidemic outbreak for *VC* ($R_0 = 1$) are

$$VC^* = \frac{1}{T_h} \text{ or 1/infectious period.} \qquad \text{(S4)}$$

The infectious period of dengue is chosen to be the same as the viremic period since the duration of infectiousness in individual humans has not been evaluated. It has only been estimated collectively from cohorts of humans, as a function of day of illness[20]. The viremic period is estimated from the intrinsic incubation period (IIP), the time between the onset of symptoms and the latent period (the period between infection and the onset of infectiousness), since most individuals have been noted to become infectious within a day before or after the onset of disease[21]. A range of IIP[22] was cited depending on the sources: 4–10 days from the World Health Organization[23], 3–14 days from the Centers for Disease Control and Prevention[24], but mostly 4–7 days[22,25]. Here we assume that the infectious period is between 4–10 days and centered around 5 days[20]. This means that for an epidemic outbreak to take place, the *VC* must be larger than

$$VC^* = 0.2 \ day^{-1} \text{ (or between 0.1 } day^{-1} \text{ and 0.25 } day^{-1}\text{),} \qquad \text{(S5)}$$

for the basic reproduction number R_0 to be larger than 1. The range of threshold values in the parentheses is based on the range of infectious period (4 ≤ T_h ≤ 10 days. Analysis of both the outbreak in Madeira in 2012 and the first dengue epidemic in over 70 years in Japan confirmed that DEP exceeded this threshold coinciding with the transmission[26].

S6. Sensitivity Analysis of *VC* for *Aedes* vectors

Many factors and uncertainties may affect the exact dengue transmission time and time windows for the values of *VC* to go over the threshold, such as, the threshold values of the dengue transmission from 0.1 to 2.5 day^{-1} (Eq. (S5)) and vector parameters used in Eq. (S1), to name a few. In this section, we will examine a few factors through sensitivity analysis of them to *VC* estimation.

S6.1. Uncertainty of six vector parameters to VC: Monte Carlo simulation and VC ± 95%CI

Sensitivity analysis of *VC* to its six vector parameters was carried out using Monte Carlo (MC) simulation[27]. The 95% Confidence Interval (or Credibility Intervals in MC[28]) and mean of *VC* were estimated for 19 temperature points ranging from 10 to 32.5 °C. Each vector parameter was varied randomly following a normal distribution around its mean with standard deviation (SD), σ = 5%*mean. Thus, approximately 95% of parameter values falls within ±2σ or ±10% from its mean. This choice of SD was based on the vector mortality rate, μ_m, whose variance was 6-16% from experimental data[17]. This is the only uncertainty estimate found in the

literature among all of the five independent vector parameters (*m* depends on μ_m as shown in Eq. (S2)). To make it uniform, we assumed the same SD of 5%*mean for all six vector parameters in order to evaluate the sensitivity of *VC* to their variation.

In the MC simulations, at each temperature the mean of each of the six vector parameters was calculated first based on their temperature dependent relationships. We then repeated the same procedure 1000 times under the random generation of each of the six parameters around their means. The corresponding values of *VC* were calculated based on Eq. (S1) for each of the 1000 runs. The 2.5th and the 97.5th percentiles of the *VC* and each of the six parameters were identified to give the value of 95%CI and their means. Repeating this process over the temperature range, the dependences of *VC* ± 95%CI and its six parameters on temperature were obtained. Table S2 shows the results for each vector parameter and *VC* for both *Aedes* vectors. Figure S3 shows the temperature dependence of the mean *VC* and *VC* ± 95%CI for both *Aedes* vectors. Similar tilted bell shaped temperature relations were observed for *VC* ± 95%CI as for the mean *VC* and for *Ae. albopictus* as for *Ae. aegypti*.

Table S2a

T (°C)	*n* (day)				μ_M (day^{-1})				*a*(day^{-1})				*m*				b_m				b_h				VC (day^{-1})			
	mean	n-95%CI	n+95%CI	sd	mean	μ_M- 95%CI	μ_M+95%CI	sd	mean	a-95%CI	a+95%CI	sd	mean	m-95%CI	m+95%CI	sd	mean	b_m-95%CI	b_m+95%CI	sd	mean	b_h-95%CI	b_h+95%CI	sd	mean	VC-95%CI	VC+95%CI	sd
10.0	54.41	49.07	59.95	2.78	0.092	0.083	0.101	0.0046	0.14	0.12	0.15	0.007	0.42	0.38	0.47	0.021	0.00	0.00	0.00	0.000	0.00	0.00	0.00	0.000	0.00	0.00	0.00	0.000
12.0	43.43	38.93	47.38	2.16	0.058	0.052	0.064	0.0028	0.15	0.13	0.16	0.007	0.67	0.60	0.73	0.034	0.00	0.00	0.00	0.000	0.00	0.00	0.00	0.000	0.00	0.00	0.00	0.000
14.0	34.84	31.32	38.28	1.77	0.042	0.038	0.046	0.0020	0.15	0.14	0.17	0.008	0.94	0.84	1.03	0.047	0.12	0.10	0.13	0.006	0.11	0.10	0.12	0.005	0.00	0.00	0.00	0.000
16.0	28.07	25.28	30.77	1.39	0.036	0.032	0.039	0.0018	0.16	0.15	0.18	0.008	1.09	0.99	1.18	0.051	0.26	0.24	0.29	0.013	0.25	0.23	0.28	0.013	0.02	0.01	0.03	0.003
18.0	22.82	20.67	24.92	1.11	0.035	0.032	0.039	0.0018	0.17	0.15	0.19	0.008	1.10	1.00	1.21	0.055	0.41	0.37	0.45	0.020	0.41	0.37	0.45	0.020	0.07	0.05	0.09	0.011
20.0	18.74	16.94	20.57	0.94	0.036	0.032	0.040	0.0019	0.18	0.16	0.20	0.009	1.08	0.97	1.19	0.054	0.55	0.50	0.61	0.027	0.57	0.52	0.62	0.028	0.15	0.11	0.21	0.025
22.0	15.53	14.05	16.98	0.76	0.036	0.033	0.040	0.0017	0.19	0.17	0.21	0.009	1.08	0.98	1.19	0.053	0.70	0.63	0.77	0.036	0.72	0.66	0.80	0.035	0.31	0.22	0.41	0.049
24.0	13.01	11.75	14.23	0.64	0.034	0.031	0.037	0.0017	0.20	0.18	0.22	0.010	1.15	1.04	1.27	0.057	0.85	0.76	0.93	0.043	0.86	0.77	0.94	0.043	0.62	0.46	0.82	0.092
25.0	11.97	10.92	13.13	0.58	0.032	0.029	0.035	0.0016	0.20	0.18	0.22	0.010	1.22	1.10	1.35	0.061	0.92	0.83	1.01	0.046	0.91	0.82	0.99	0.046	0.88	0.65	1.16	0.131
26.0	11.03	10.00	12.09	0.55	0.030	0.027	0.033	0.0016	0.21	0.19	0.22	0.010	1.30	1.17	1.42	0.066	0.99	0.90	1.09	0.050	0.95	0.85	1.04	0.048	1.23	0.92	1.63	0.180
27.0	10.20	9.19	11.17	0.50	0.028	0.025	0.031	0.0014	0.21	0.19	0.23	0.011	1.38	1.25	1.52	0.068	1.00	0.90	1.10	0.050	0.97	0.88	1.06	0.048	1.59	1.15	2.08	0.236
28.0	9.52	8.62	10.43	0.49	0.027	0.024	0.029	0.0013	0.21	0.19	0.23	0.011	1.47	1.32	1.61	0.073	1.00	0.90	1.10	0.052	0.97	0.88	1.06	0.048	1.91	1.41	2.51	0.294
29.0	8.88	8.03	9.77	0.45	0.026	0.023	0.028	0.0013	0.22	0.20	0.24	0.011	1.50	1.34	1.66	0.077	1.00	0.91	1.10	0.049	0.94	0.84	1.03	0.048	2.07	1.52	2.70	0.311
30.0	8.31	7.52	9.10	0.41	0.027	0.024	0.030	0.0013	0.22	0.20	0.24	0.011	1.45	1.32	1.59	0.073	1.00	0.90	1.10	0.052	0.87	0.79	0.95	0.043	1.87	1.38	2.43	0.276
30.7	7.94	7.20	8.75	0.40	0.029	0.026	0.032	0.0015	0.23	0.20	0.25	0.011	1.36	1.23	1.49	0.066	1.00	0.90	1.09	0.050	0.78	0.71	0.86	0.039	1.52	1.14	1.98	0.214
31.4	7.62	6.90	8.37	0.38	0.032	0.029	0.035	0.0016	0.23	0.21	0.25	0.011	1.23	1.10	1.34	0.061	1.00	0.90	1.09	0.049	0.65	0.58	0.71	0.031	1.02	0.76	1.34	0.150
32.0	7.38	6.65	8.09	0.36	0.036	0.032	0.039	0.0018	0.23	0.21	0.25	0.012	1.09	0.98	1.20	0.056	1.00	0.90	1.10	0.052	0.45	0.40	0.49	0.022	0.57	0.42	0.73	0.083
32.3	7.24	6.53	7.95	0.36	0.038	0.034	0.042	0.0019	0.23	0.21	0.25	0.012	1.02	0.92	1.12	0.050	1.00	0.89	1.10	0.053	0.27	0.24	0.30	0.014	0.30	0.22	0.39	0.044
32.5	7.18	6.49	7.85	0.35	0.040	0.036	0.044	0.0020	0.23	0.21	0.26	0.012	0.98	0.88	1.08	0.052	1.00	0.90	1.10	0.051	0.00	0.00	0.00	0.000	0.00	0.00	0.00	0.000

Table S2b

T (°C)	*n* (day)				μ_M (day^{-1})				*a*(day^{-1})				*m*				b_m				b_h				VC (day^{-1})			
	mean	n-95%CI	n+95%CI	sd	mean	μ_M- 95%CI	μ_M+95%CI	sd	mean	a-95%CI	a+95%CI	sd	mean	m-95%CI	m+95%CI	sd	mean	b_m-95%CI	b_m+95%CI	sd	mean	b_h-95%CI	b_h+95%CI	sd	mean	VC-95%CI	VC+95%CI	sd
10.0	54.41	49.07	59.95	2.78	0.026	0.023	0.028	0.0013	0.15	0.13	0.16	0.007	1.32	1.19	1.45	0.067	0.00	0.00	0.00	0.000	0.00	0.00	0.00	0.000	0.00	0.00	0.00	0.000
12.0	43.43	38.93	47.38	2.16	0.023	0.021	0.025	0.0011	0.10	0.09	0.10	0.005	1.50	1.35	1.64	0.075	0.00	0.00	0.00	0.000	0.00	0.00	0.00	0.000	0.00	0.00	0.00	0.000
14.0	34.84	31.32	38.28	1.77	0.024	0.022	0.026	0.0012	0.07	0.06	0.08	0.004	1.43	1.28	1.57	0.071	0.12	0.10	0.13	0.006	0.08	0.07	0.08	0.004	0.00	0.00	0.00	0.000
16.0	28.07	25.28	30.77	1.39	0.027	0.025	0.030	0.0013	0.07	0.06	0.08	0.003	1.27	1.15	1.38	0.060	0.26	0.24	0.29	0.013	0.18	0.16	0.19	0.009	0.00	0.00	0.01	0.001
18.0	22.82	20.67	24.92	1.11	0.031	0.028	0.034	0.0015	0.08	0.07	0.09	0.004	1.13	1.02	1.23	0.056	0.41	0.37	0.45	0.020	0.29	0.26	0.31	0.014	0.01	0.01	0.02	0.002
20.0	18.74	16.94	20.57	0.94	0.033	0.030	0.036	0.0017	0.11	0.10	0.12	0.005	1.04	0.94	1.14	0.052	0.55	0.50	0.61	0.027	0.40	0.36	0.44	0.020	0.05	0.03	0.06	0.007
22.0	15.53	14.05	16.98	0.76	0.034	0.031	0.037	0.0016	0.14	0.13	0.16	0.007	1.01	0.91	1.11	0.050	0.70	0.63	0.77	0.036	0.50	0.46	0.56	0.025	0.13	0.09	0.17	0.020
24.0	13.01	11.75	14.23	0.64	0.033	0.030	0.037	0.0016	0.18	0.16	0.20	0.009	1.03	0.92	1.13	0.051	0.85	0.76	0.93	0.043	0.60	0.54	0.66	0.030	0.33	0.24	0.43	0.049
25.0	11.97	10.92	13.13	0.58	0.033	0.030	0.036	0.0016	0.20	0.18	0.22	0.010	1.05	0.95	1.16	0.053	0.92	0.83	1.01	0.046	0.64	0.57	0.69	0.032	0.50	0.37	0.65	0.074
26.0	11.03	10.00	12.09	0.55	0.032	0.029	0.035	0.0016	0.21	0.19	0.23	0.010	1.08	0.98	1.18	0.055	0.99	0.90	1.09	0.050	0.66	0.60	0.73	0.034	0.72	0.54	0.96	0.105
27.0	10.20	9.19	11.17	0.50	0.031	0.028	0.034	0.0015	0.23	0.21	0.25	0.012	1.11	1.01	1.22	0.055	1.00	0.90	1.10	0.050	0.68	0.61	0.74	0.034	0.93	0.68	1.23	0.140
28.2	9.39	8.50	10.29	0.48	0.030	0.027	0.033	0.0015	0.24	0.22	0.26	0.012	1.15	1.04	1.26	0.057	1.00	0.90	1.10	0.052	0.68	0.61	0.74	0.034	1.16	0.86	1.52	0.179
29.3	8.71	7.87	9.57	0.44	0.029	0.026	0.032	0.0015	0.25	0.23	0.27	0.012	1.16	1.04	1.29	0.060	1.00	0.91	1.10	0.049	0.65	0.58	0.71	0.033	1.25	0.92	1.63	0.189
30.2	8.20	7.43	8.99	0.40	0.030	0.027	0.033	0.0015	0.25	0.23	0.28	0.012	1.15	1.05	1.27	0.058	1.00	0.90	1.10	0.052	0.59	0.54	0.65	0.030	1.17	0.86	1.53	0.174
31.0	7.80	7.07	8.60	0.40	0.031	0.028	0.034	0.0016	0.26	0.23	0.28	0.013	1.12	1.02	1.23	0.054	1.00	0.90	1.09	0.050	0.51	0.46	0.56	0.025	0.97	0.73	1.26	0.137
31.5	7.58	6.86	8.32	0.38	0.032	0.028	0.035	0.0016	0.25	0.23	0.28	0.013	1.09	0.98	1.19	0.054	1.00	0.90	1.09	0.049	0.43	0.39	0.48	0.021	0.76	0.56	0.99	0.111
32.0	7.38	6.65	8.09	0.36	0.033	0.030	0.036	0.0016	0.25	0.23	0.27	0.013	1.05	0.94	1.15	0.054	1.00	0.90	1.10	0.052	0.31	0.28	0.34	0.015	0.50	0.37	0.64	0.073
32.3	7.24	6.53	7.95	0.36	0.034	0.030	0.037	0.0017	0.25	0.22	0.27	0.012	1.02	0.92	1.11	0.050	1.00	0.89	1.10	0.053	0.19	0.17	0.21	0.010	0.27	0.21	0.36	0.040
32.5	7.18	6.49	7.85	0.35	0.034	0.031	0.038	0.0018	0.25	0.22	0.27	0.012	1.00	0.90	1.10	0.053	1.00	0.90	1.10	0.051	0.00	0.00	0.00	0.000	0.00	0.00	0.00	0.000

Table S2. Temperature dependent *VC* and its six vector parameters with 95% CI using *Monte Carlo* simulation for (a) *Ae. aegypti* (b) *Ae. albopictus*. The 95% Credibility Intervals and mean were estimated for each temperature ranging from 10 to 32.5. Each vector parameter was varied by σ = 5% from its mean using normal random number generator. The total number of runs was 1000.

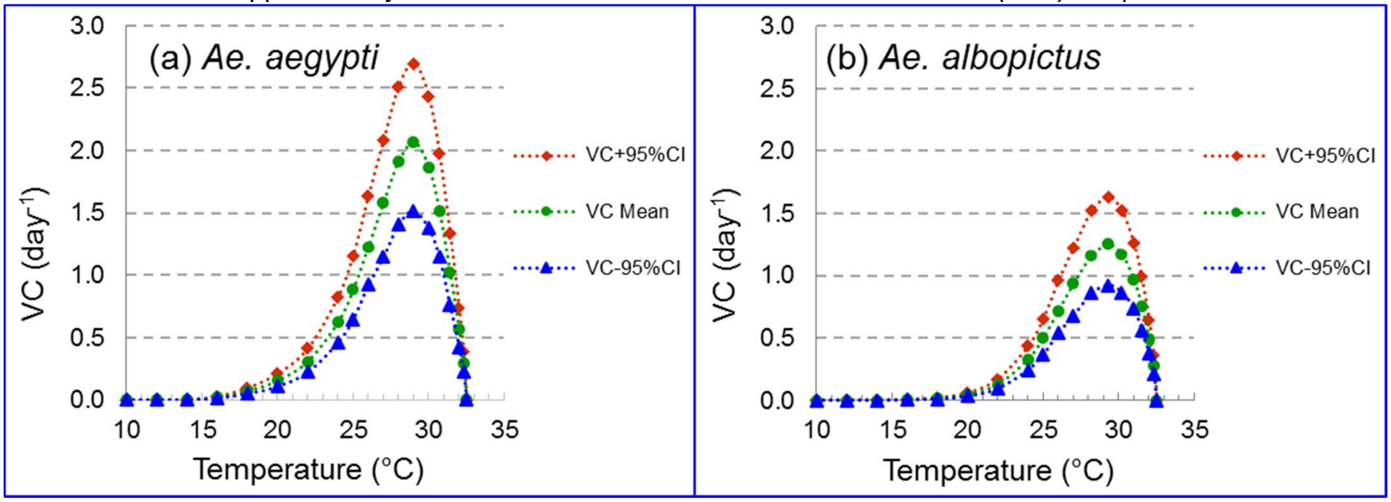

Figure S3. Sensitivity Analysis of *VC* to the variation of its six vector parameters using Monte Carlo simulation for (a) *Ae. aegypti* and (b *Ae. albopictus*. The 95% Confidence Interval and mean of *VC* were estimated for each temperature ranging from 10 to 32.5. Each vector parameter was varied by σ = 5% around its mean using normal random number generator. The total number of runs was 1000.

S6.2. VC ± 95%CI for ten European cities over three periods of the 21st century

Fitting the *VC* ± 95%CI data in Table S2 with 5th order polynomial functions, analytical relationships for *VC* ± 95%CI with temperature were obtained. Using these fitted functions and Eq. (S1) and temperature data as input, we estimated *VC* ± 95%CI and *VC* for ten European cities over three periods during this century. Figure S4 shows the results on seasonality of dengue epidemic potential for two *Aedes* vectors: *Ae. aegypti* (Fig. S4A) and *Ae. albopictus* (Fig. S4B). In each figure, the top row corresponds to the averaged *VC* over the recent decade (2004-2013 using CRU-TS3.22[1] temperature data); the middle and bottom rows to three decadal averaged *VC* over the middle (2030-2059) and the end (2070-2099) of this century. The corresponding estimates for the future were calculated using projected future temperatures (see Methods section for details) under the highest greenhouse gas emission pathway (RCP 8.5)[29] where *VC* were averaged over 5 projected climate models (CMIP5[36]). In these calculations, DTR was included and m_{max} =1.5. In each figure, the middle column (Figure S4 (ii, v, viii)) corresponds to the mean values of *VC* where Figure S4(ii) and S4(viii) are the same as Figure 2 (only European 10 cities) and 3(vi) in the main article; the left column (Figure S4 (i, iv, vii)) corresponds to *VC* + 95%CI – the upper limit and the right column (Figure S4 (iii, vi, ix)) to *VC* - 95%CI — the lower limit.

As shown in Fig. S4, the same seasonality of *VC* is observed in all three curves: *VC* mean and *VC*± 95%CI, although the width and height varies from its mean. The same characteristic transmission window and intensity for dengue epidemic potential were observed under both limits of uncertainty and for both vectors. Over the recent decade (Fig. S4 top row), for *Ae. aegypti* six cities (using *VC*-95%CI) to seven cities (using VC or *VC*+95%CI) among the ten estimated were over the threshold (0.2 day^{-1}) for at least one month of the year in dengue epidemic transmission; for *Ae. albopictus*, they were three cities (using *VC*-95%CI) to five cities (using *VC*+95%CI) in Europe among the ten estimated were over the threshold.

In the future under the worst case scenario of RCP8.5, for *Ae. aegypti* all of the 10 cities will be over the dengue transmission threshold for at least one month of the year within the limits of 95%CI, although the duration will be different depending on the time period, the upper and the lower limit of 95%CI; for *Ae. albopictus*, the number of cities that is over the dengue transmission threshold would be six (using *VC*-95%CI) to eight cities (using *VC*+95%CI) in the middle of this century (2030-2059), and all of the 10 cities by the end of this century within the limits of 95% CI.

Therefore, there will be no qualitative changes in the conclusions drawn from Fig. 2-3 after considering the uncertainty of vector parameters. It is a matter of when and the exact number of months in duration (or time windows) over which this would happen. Our study has shown a clear upward trend in dengue epidemic potential over the 200 years' time and seasonality transmission window during the year. This holds even after vector parameters' uncertainties were included.

Figure S4A. *VC ± 95%CI for Ae. aegypti*

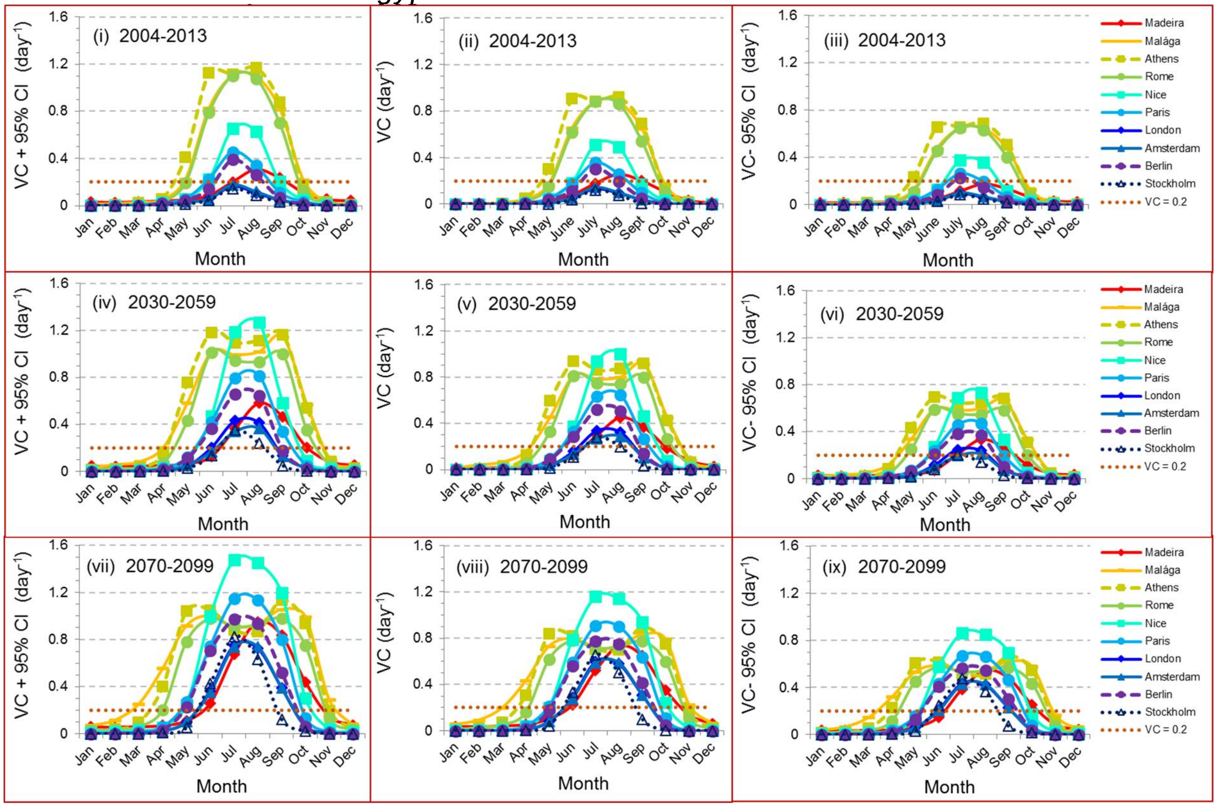

Figure S4B. *VC ± 95%CI for Ae. albopictus*

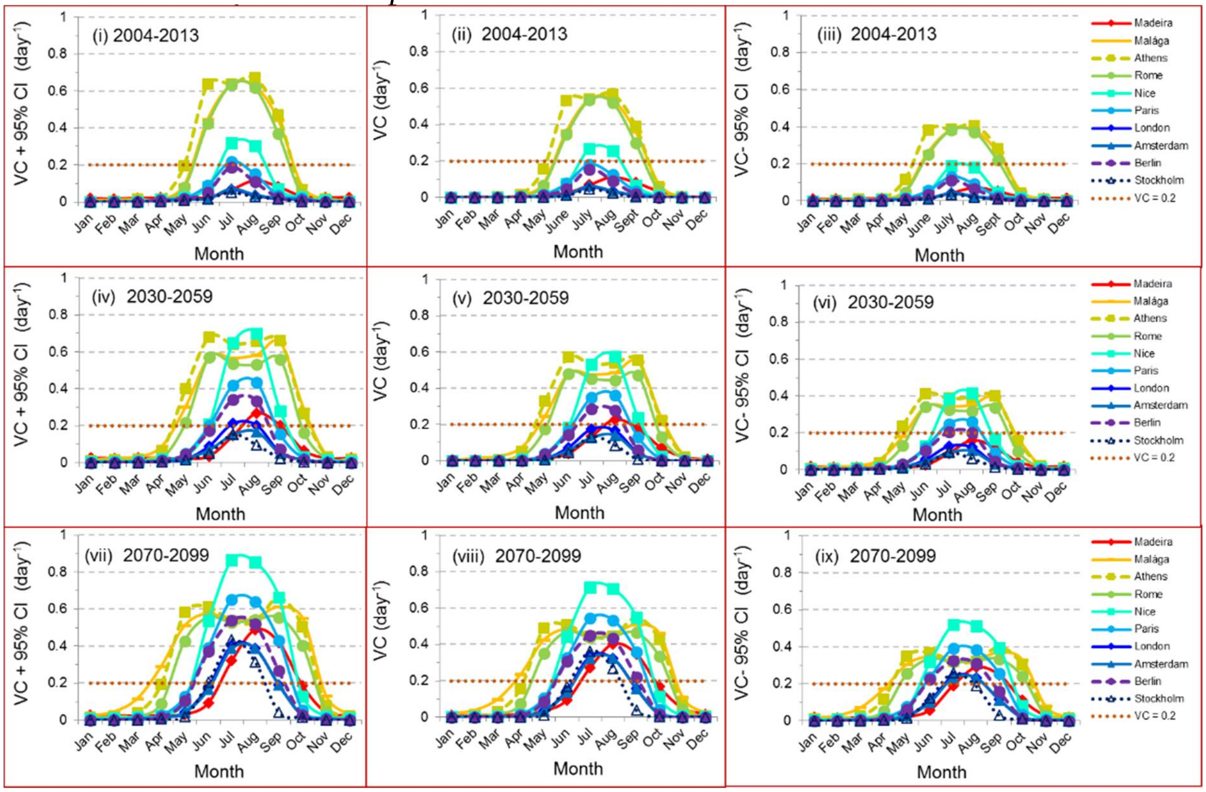

Figure S4. Comparison of seasonality curves for ten European cities between the mean of *VC* and the *VC*± 95% CI for *Ae. aegypti* (Figure S4A) and *Ae. albopictus* (Figure S4B). The *VC* in Fig. S4 (B, E, H) were calculated from Eq. (S1) and *VC* ± 95% CI in the rest of figures were estimated from Fig. S3. All *VC* values were averaged over either one decade (top row) using CRU-TS3.22[3] temperature data or over three decades (the middle and low rows) and 5 models (CMIP5[36]) using projected future temperature data under RCP8.5. DTR was included with m_{max} =1.5.

S6.3. Uncertainty of threshold (infectious period) to dengue transmission duration

So far all the estimation and discussion in the main article for transmission window (Fig. 4) was based on the epidemic outbreak threshold of $VC^*= 0.2\ day^{-1}$, which was based on the assumption of the infectious period of 5 days[20]. As stated in Section S5, the infectious period for dengue is between 4 and 10 days. This means that the threshold of *VC* for the dengue epidemic outbreak is between 0.1 and 0.25 day^{-1} as shown in Equation (S5). The uncertainty of infectious period and therefore the threshold value affects the transmission window shown in Fig. 4. Using the upper and lower limit of the threshold conditions, we estimated the number of months that *VC* would be over the threshold for the rest of the century.

Figure S5 shows effect of infectious period on the duration (number of months) of dengue epidemic potential when decadally averaged *VC* will be over the threshold during the nine decades of this century. Two climate scenarios were estimated: RCP2.6 (top row) and RCP8.5 (bottom row) for two vectors: *Ae. aegypti* (Fig. S5A) and *Ae. albopictus* (Fig. S5B). Three different threshold values (VC^*=0.1, 0.2, and 0.25 day^{-1}) were compared which corresponded to three infectious periods (T_h): 4 (left columns in Fig. 5), 5 (middle) and 10 (right) days within the infectious period range of 4-10 days.

Under the best climate scenario (RCP2.6), for *Ae. aegypti* the number of cities that would have consecutive three or more months over the threshold ranges from 4, 6 to 7 cities when using the threshold condition from the lower, middle to the upper limits; for *Ae. albopictus* they are 3, 3 to 4 cities over the threshold corresponding to the lower to the upper threshold limits.

Under the worst climate scenario (RCP8.5), for *Ae. aegypti* the number of cities that would have consecutive three or more months over the threshold ranges from 7, 9 to 10 cities when using the threshold condition from the lower, middle to the upper limits; for *Ae. albopictus* they are 5, 6 to 9 cities over the threshold corresponding to the lower to the upper threshold limits.

Thus, our conclusion based on Fig. 4 is unchanged since we have chosen a strict threshold condition – 0.2 day^{-1} which is very close to the upper limit of 0.25 day^{-1}. However, the decade that Nice is over the threshold is 2011s under the threshold condition of 0.2 day^{-1} (Fig. 4a (B)), which would be delayed by one decade to 2020s (Fig. S5a (left)) under the upper limit of the threshold condition: 0.25 day^{-1} (T_h = 4 days). This comparison among the three columns in Fig. S5 shows importance of the threshold condition based on the infectious period. We have chosen conservatively.

Figure S5A. Transmission window vs. infectious period (T_h) - *Ae. aegypti*

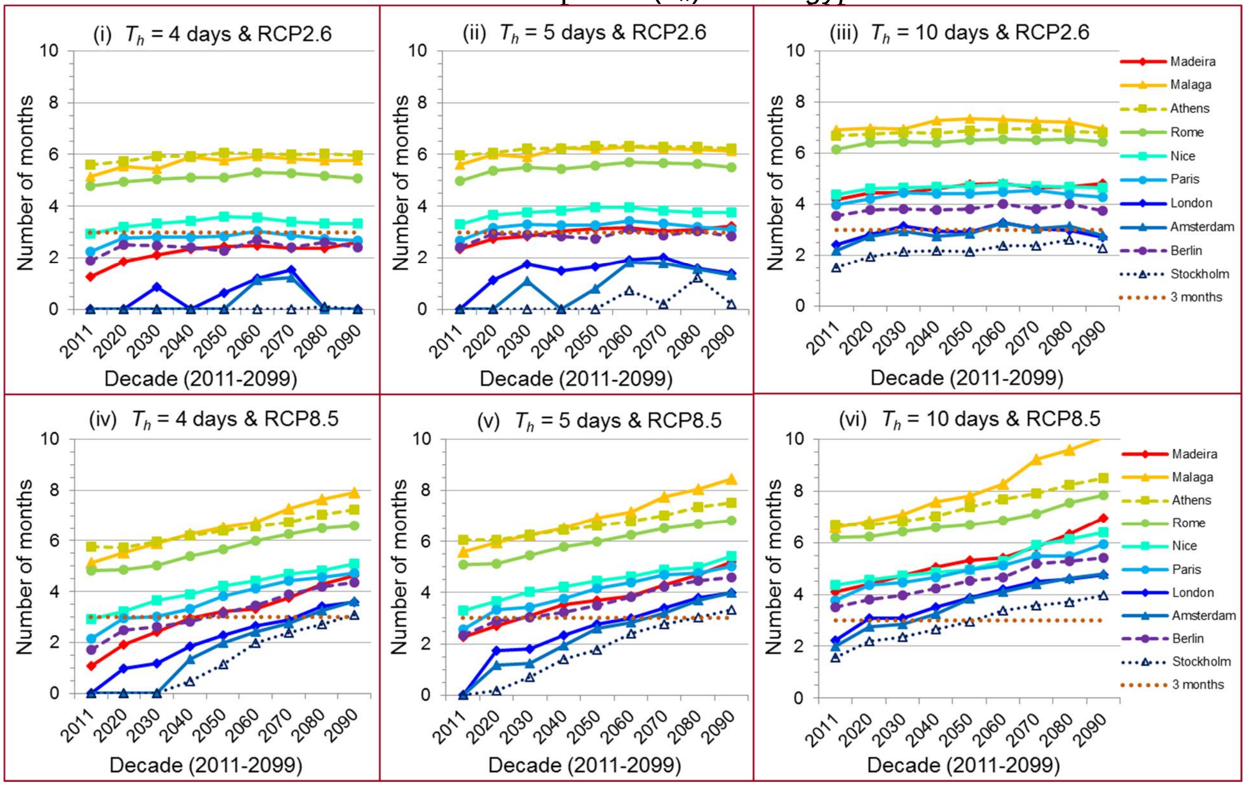

Figure S5B. Transmission window vs. infectious period (T_h) - *Ae. albopictus*

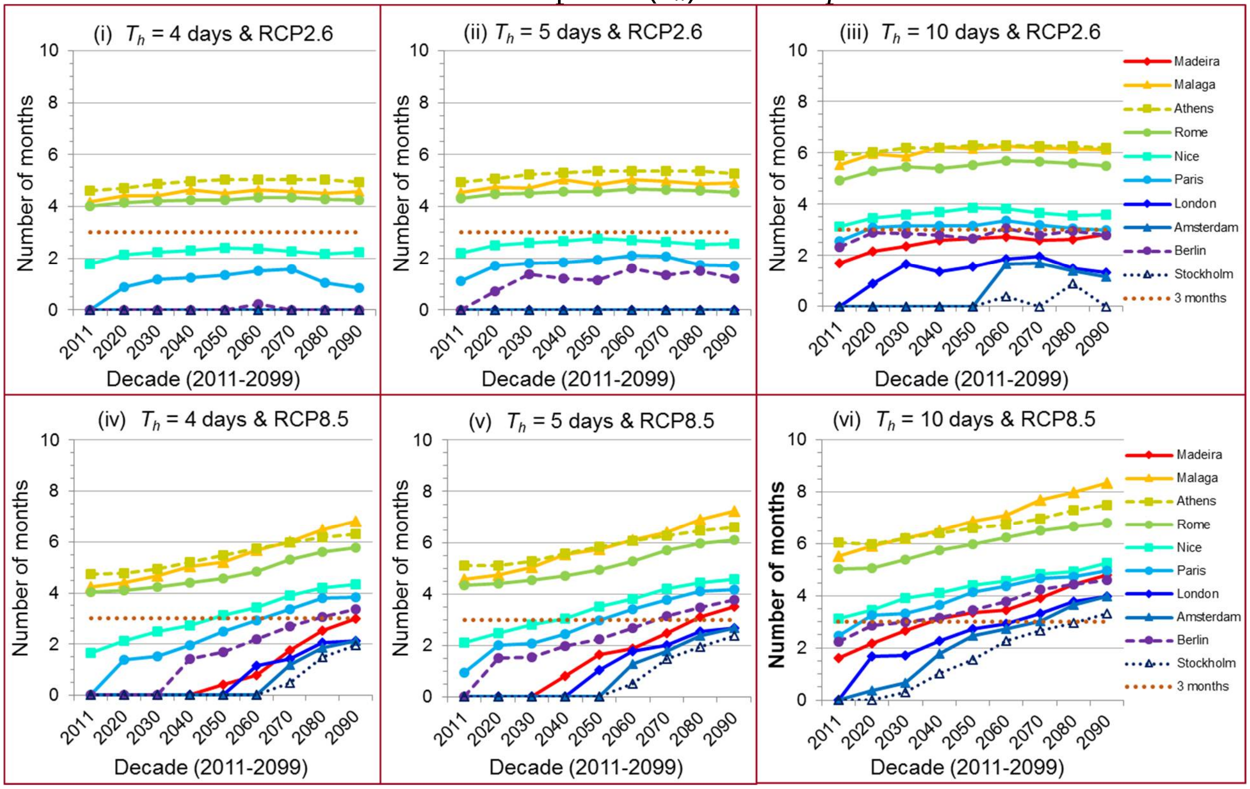

Figure S5. The number of months that the *VC* is over threshold based on uncertainty of the infectious period (T_h) for *Ae. aegypti* (Fig. S5A) and *Ae. albopictus* (Fig. S5B). Left column: the upper limit of the threshold (VC^*=0.25 day^{-1}) based on the shortest infectious period of 4 days; middle column: the typical threshold used in the main paper (VC^*=0.2 day^{-1}) based on the infectious period of 5 days; right column: the lower limit of the threshold (VC^*=0.1 day^{-1}) based on the longest infectious period of 10 days. Top curves correspond to the best climate scenario of RCP2.6 and the bottom the worst climate scenario of RCP8.5 over nine decades during this century. DTR was included and m_{max}=1.5.

S6.4. Uncertainty of vector population (m_{max}) to dengue transmission intensity and duration

VC depends on temperature through its six vector parameters as shown in Equation (S1). Among them, five temperature dependent relationships were obtained through literature. The female vector-to-human population ratio, *m*, were unknown. Assumption on how *m* depended on temperature was made as shown in Eq. (S2) where a constant c were chosen in such a way so that m_{max}=1.5. The uncertainty of m_{max} is addressed here. We varied m_{max} from 1 to 2 and estimated *VC* for both the past and future decades and two dengue vectors.

Figure S6 shows the effect of maximum female vector-to-human population ratio (m_{max}) on the seasonality of *VC* for 13 selected cities that correspond to Fig. 2 in the main article. Here *VC* were averaged over the recent decade (2004 – 2013) using CRU-TS3.22 monthly temperature data and DRT was included. The top row is for *Ae. aegypti* and the bottom row is for *Ae. albopictus*. Three values of m_{max} were compared: m_{max} = 2 (left column), 1.5 (middle – same as Fig. 2) and 1 (right column). Similar shapes in seasonality curves were observed for all three values of m_{max} with different peak intensity as expected from the linear relationship of *VC* and *m* (Eq. (S1)). *Ae. albopictus* showed lower magnitude of *VC* than *Ae. aegypti* but the same effect on seasonality curves from different m_{max} values.

As in the Fig. 4 in the main article, we have defined 1) dengue transmission intensity as the decadally averaged *VC* over the highest three consecutive months of the year, and 2) transmission window or duration as the number of months that the decadally averaged *VC* is over the threshold. Figure S7 shows the effect of varying m_{max} on the dengue transmission intensity over two centuries for ten European cities under two RCPs. Fig. S7A is the estimation for *Ae. aegypti* and Fig. S7B for *Ae. albopictus*. The top row is estimation of intensity under RCP2.6 and the bottom row under RCP8.5. Three values of m_{max} were compared in Fig. S7: m_{max} = 2 (left column), 1.5 (middle – same as Fig. 4 (A)) and 1 (right column). The higher the m_{max} values, the higher the transmission intensity. The higher the greenhouse gas emission pathway (RCP), the higher the intensity and the speed of intensity increase over this century. Under RCP2.6, the dengue transmission intensity will level off during the next few decades regardless the vector population. However, reducing the female vector population will bring some Northern to central European cities from above to under the threshold for dengue epidemics. Under RCP8.5, the dengue transmission intensity will keep increase from 1970s till end of this century with a speed and maximum value depending on the m_{max} values: the higher the m_{max} value, the faster the speed of increase in transmission intensity and the higher the maximum transmission intensity at the end of this century.

Figure S6. Seasonality of *VC* vs. vector population for both *Aedes* vectors

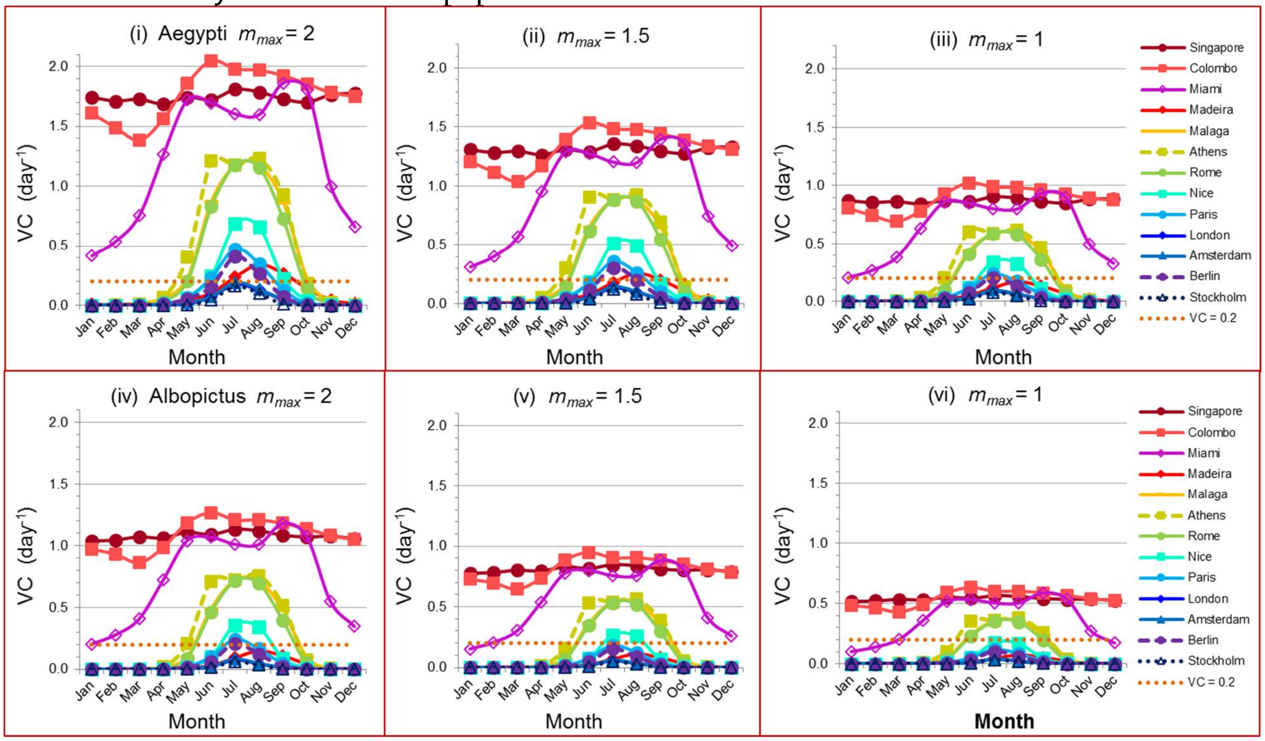

Figure S6. Effect of maximum female vector-to-human population ratio (m_{max}) on the seasonality of *VC* for 13 selected cities – ten European cities plus three reference cities from both tropical and subtropical areas. Top row is for *Ae. aegypti* and bottom row is for *Ae. albopictus*. Here *VC* is averaged over the recent decade (2004-2013) for each month of the year. DTR is included. CRU-TS3.22 monthly temperature data was used.

Figure S7A. Dengue transmission intensity vs. m_{max} for *Ae. aegypti*

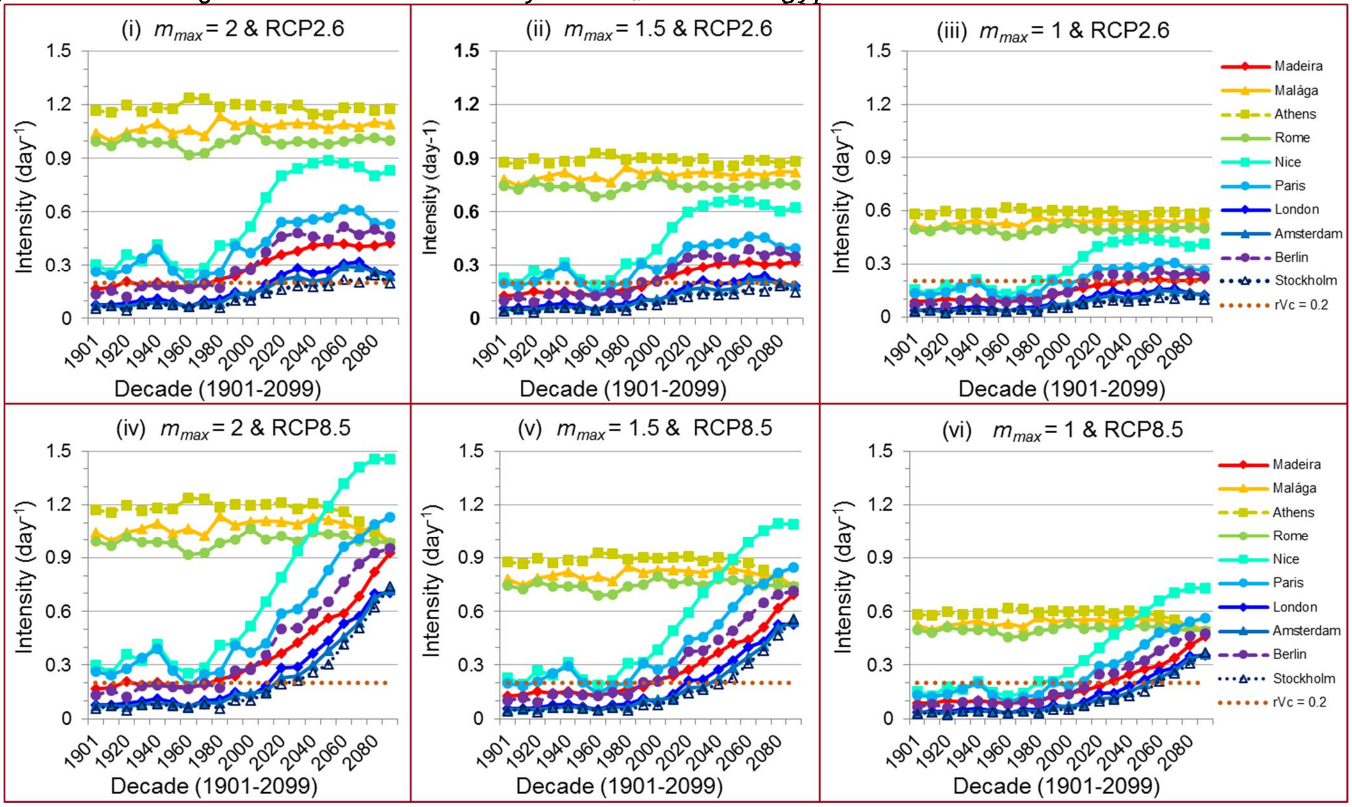

Figure S7B. Dengue transmission intensity vs. m_{max} for *Ae. albopictus*

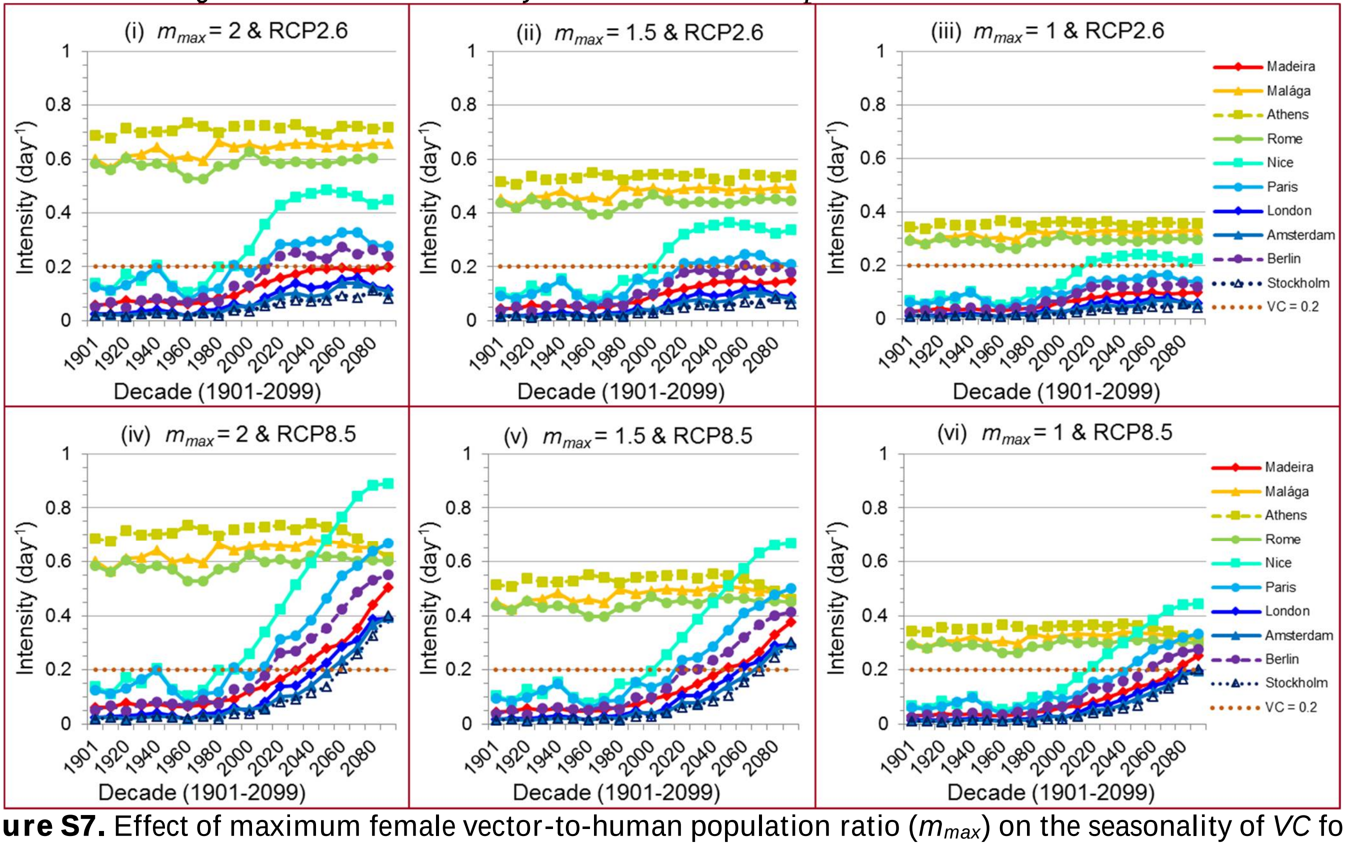

Figure S7. Effect of maximum female vector-to-human population ratio (m_{max}) on the seasonality of *VC* for 10 selected European cities over two centuries for *Ae. aegypti* (Fig. S7A) and for *Ae. albopictus* (Fig. S7B). Historical temperatures were used from 1901 to 2009. From 2011 to 2099, two emission pathways were evaluated: RCP2.6 (i-iii) and RCP8.5 (iv-vi). DTR is included. Intensity was defined as the averaged *VC* over the highest consecutive 3-months for each decade.

Figure S8 shows the effect of varying m_{max} on the dengue transmission window over two centuries for ten European cities. The transmission duration that decadally averaged *VC* is over the threshold value of 0.2 day^{-1} was estimated for *Ae. aegypti* (Fig. S8A) and *Ae. albopictus* (Fig. S8B) under RCP2.6 (top row) and RCP8.5 (bottom row). As in Figure S6 & S7, three values of m_{max} were compared in Fig. S8: m_{max} = 2 (left column), 1.5 (middle – same as Fig. 4 (B)) and 1 (right column). The higher the m_{max} values, the longer the transmission duration. The higher the greenhouse gas emission pathway (RCP), the longer the transmission duration and the speed of transmission duration increase over this century. Like the transmission intensity, under RCP2.6, the dengue transmission window will level off during the next few decades regardless the vector population. However, reducing the female vector population from m_{max} = 2 to 1 will bring all the central European cities for *Ae. aegypti* and Nice for *Ae. albopictus* from above to under the threshold for dengue epidemics if using three months duration as the threshold. In contrast, under RCP8.5, the dengue transmission duration will keep increasing from 1970s to the end of this century such that the time for the dengue epidemic to occur becomes earlier as m_{max} increases.

From the sensitivity analysis of maximum female vector-to-human population ratio to transmission intensity and duration, it is clear that vector population is important in the dengue epidemic potential estimation. Without the vector, there will be no dengue transmission. Reducing female vector population can bring the dengue epidemic potential intensity and duration from over to under the threshold. It can also delay the outbreak occurring time. This is especially important in the later of this century for those cities whose *VC* values are above but close to the threshold such as the Southern to Central Europe for *Ae. albopictus* and Northern (RCP8.5) to Central Europe (all RCPs) for *Ae. aegypti*. Therefore, in the short term of next decade, vector control is very important especially in Southern Europe for *Ae. albopictus* and Southern to Central Europe for *Ae. aegypti* under RCP2.6 to stop the dengue epidemics. However, reducing global warming to change from RCP8.5 to RCP2.6 has dramatic longer term effect on dengue epidemic potential especially toward later part of this century.

For dengue outbreaks to actually occur, it requires that many necessary factors fall in place. Adequate vector population is one of them. Human exposure to mosquito biting is another. In addition, rainfall and humidity[30] are not included in this model, nor is the local environment carrying capacity. Using more climate variables would make a better projection for future dengue risk as shown in the statistical model based on empirical dengue data[31]. Other conditions, such as microclimate and human lifestyle and interventions also contribute to the proliferations of the vector population. Future studies may seek to establish further understanding of these drivers. Dengue epidemic potential can be projected better if a dengue vector population distribution related to temperature becomes available. So does more vector parameters for *Ae. albopictus*.

Dengue transmission is a complex process and not all factors needed for projecting long term transmission trends can be included in every model. We hope that our study brings one step closer in understanding the dengue epidemic potential for Europe, especially the seasonality aspect of the transmission possibility. For the dengue control, the result of this study may help policy makers to reduce cost by focusing on certain time period and certain geographical location in Europe. Additionally, our study offers vital new evidence that dengue epidemic potential across Europe increases sharply under *business as usual* emissions scenarios during the 21st century. This information should underscore the need for collective action to reduce emissions and mitigate climate change given the increasing evidence that failure to act harms human health.

Figure S8A. Dengue transmission window vs. m_{max} for *Ae. aegypti*

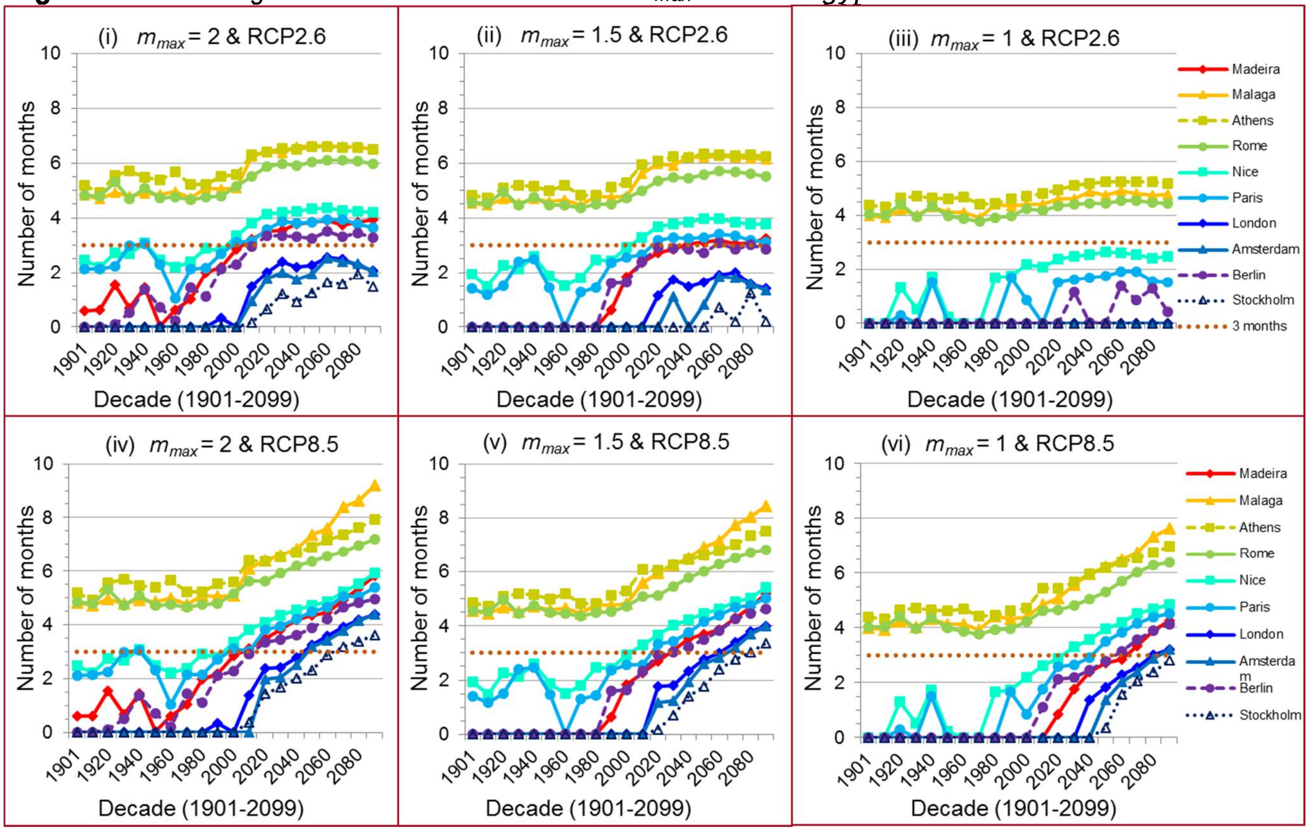

Figure S8B. Dengue transmission window vs. m_{max} for *Ae. albopictus*

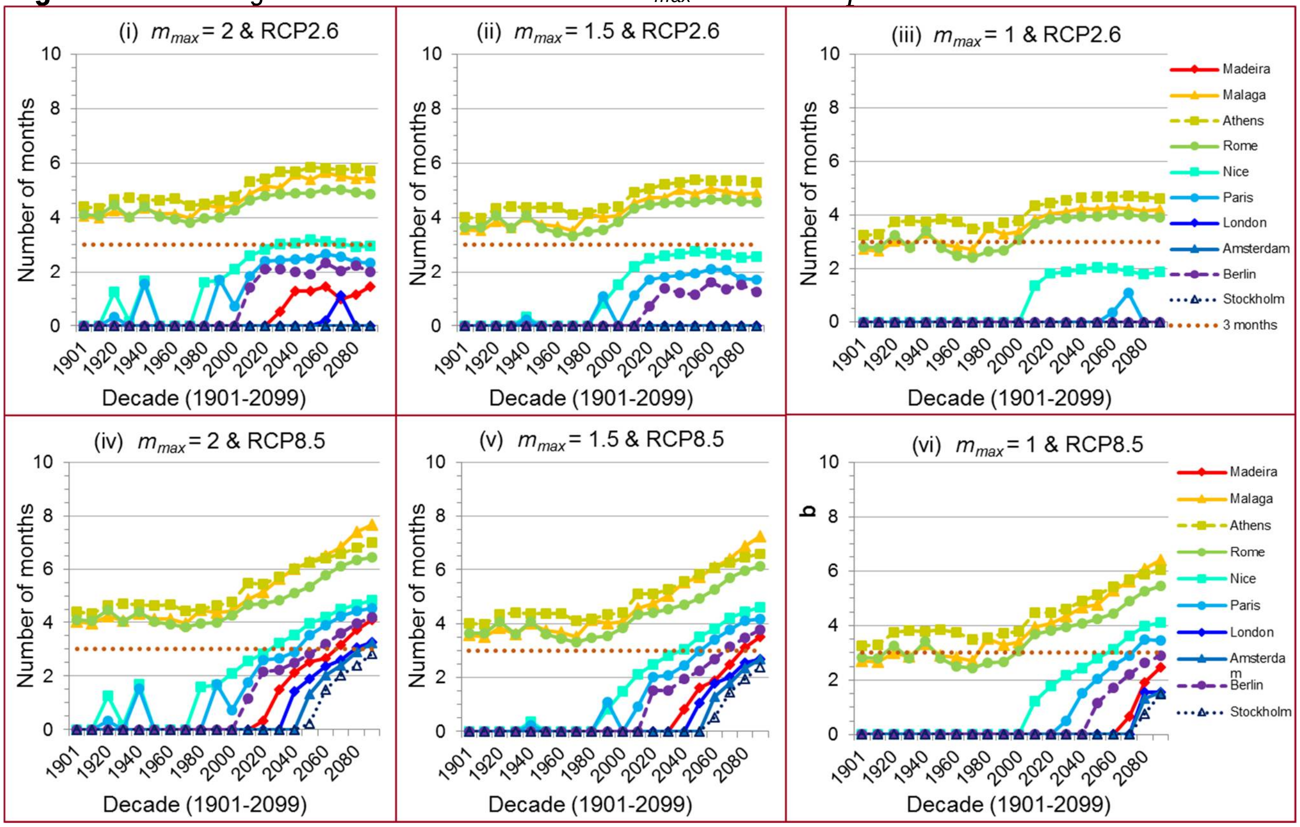

Figure S8. Effect of maximum female vector-to-human population ratio (m_{max}) on the transmission window of *VC* for 10 selected European cities over two centuries for *Ae. aegypti* (Fig. S8A) and for *Ae. albopictus* (Fig. S8B). Historical temperatures were used from 1901 to 2009. From 2011 to 2099, two emission pathways were evaluated: RCP2.6 (top) and RCP8.5 (bottom). DTR is included. Transmission window was defined as the number of months that the decadally averaged *VC* was over the threshold value of 0.2 day^{-1}.

S7. Comparison of this study with other statistical models and our previous work

S7.1. Mathematical model vs Statistical model, comparing with Bouzid et al's dengue risk mapping in Europe

Our results based on *VC,* the threshold part of a mathematical model, may be compared to the recent study on dengue risk mapping in Europe using statistical model. Bouzid, et al.[32] estimated climate change impacts on dengue fever incidence for Europe under one climate scenario – A1B, the rapid economic growth scenario (similar to RCP 8.5) to give a three snapshots of dengue risk during this century. Their future projection is based on past data - dengue cases, socioeconomic factors and climate (temperature, precipitation and humidity) from Mexico (1985-2007) and future climate data for Europe. Dengue cases in Mexico is mainly driven by *Ae. aegypti*. Thus their prediction is suited for future conditions similar to the past in Mexico for the transmission of vector mainly *Ae. aegypti*. Like our model they have overestimated the dengue risk in the past. On the other hand, they showed slow increase in the future whereas we showed greater potential increase under RCP8.5. We modeled the potential commencement of the dengue outbreaks while they modeled the actual incidence risk. We estimated *VC* for both *Aedes* vectors under four climate scenarios with decadal resolution over two centuries. Each has strength and weakness. Statistical modeling usually includes more climate variables[33,34]. Mathematical modeling is mechanism driven and tends to focus on the main factors and make assumptions on less important factors. Therefore, Mathematical modeling is less accurate for a particular area but is better to generalize geographically and temporally to situations less studied than statistical models. On the other hand, statistical modeling is association and comparison (exposure-response) based and tends to take into all the possible variables to describe a particular situation. They are more accurate to represent the past dengue areas but less accurate for the future on less studied areas. As stated by a recent review study of statistical modeling of dengue risk mapping, "Approaches using mechanistic models rather than statistical approaches…may provide more appropriate tools to explore this field of research …"[35]. Although using different approaches, our findings have high correlation spatially with their findings in Southern Europe. Both predicted increase of dengue transmission in the future. Although we have estimated higher DEP in the Central to Northern Europe, we have drawn similar conclusions – climate change is likely to increase the dengue risk in the future. Therefore, both approaches are valuable and complement each other. Readers should take both views in consideration for the future of dengue risk in Europe.

S7.2. Comparing with our previous study using relative VC for dengue epidemic potential

In this study, we have calculated temperature dependent vectorial capacity for the European extent based on past, current, and 5 models future projections for both dengue vectors and 4 climate scenarios. Especially,

1) we have expanded the model from *Aedes aegypti* to *Aedes albopictus* and displayed our finding for areas of Europe known to have *Aedes* vector populations;
2) we have incorporated greater temporal resolution to our analysis (daily, monthly, seasonal, decadal vs. 30 year averages) and resultant outputs;
3) we have incorporated greater spatial resolution refined our cartographic outputs for the European extent;
4) we have created time-space model applications of the original deterministic model and generated sensitivity analysis of each parameter involved in the *VC* calculation and interpretation;
5) we have further investigated the potential areas and periods of transmission of dengue fever in Europe, looking into the results of the model in ten areas/cities of Europe.

More details are listed in Table S3.

Table S3. Lists of the difference between this study from our previous work[6].

Category	PLoS ONE 2014 article	This European paper
**1. Method**	**Calculating dengue epidemic potential (DEP)**	**Calculating dengue epidemic potential (DEP)**
**1.1 DEP is based on**	Relative vectorial capacity - *rVc*	Vectorial capacity - *VC*
**1.2 sensitivity analysis**	None	Yes, uncertainty of 4 variables to VC:
		a. Six vector parameters to VC (Monte Carlo simulation)
		b. Threshold condition due to range of infectious period
		c. Female vector-to-human population ratio
		d. Global vs local temperature data sets
**1.3 Future climate scenarios**	Greenhouse gas emission pathway - RCP8.5 (the worst scenario)	Greenhouse gas emission pathway - RCP2.6-8.5 (all 4 scenarios)
**1.4 Map resolution**	Low resolution - 0.5x0.5 degrees monthly gridded temperature data as input	Better resolution - 0.25x0.25 degrees daily gridded temperature data as input
**2. Results**	1. Effect of temperature & diurnal temperature range (DTR) on 5 vector parameters and *rVc* for *Ae. aegypti*	1. Seasonal European maps of *VC* for *Ae. aegypti & albopictus* for current & future decade under 2 RCPs
	2. Global maps of *rVc* for Ae. *aegypti* from past, present to future under RCP8.5	2. Seasonality: VC vs. Month for 10 European cities, current & future decade under 4 RCPs
		3. Transmission intensity and duration of 10 EU cities over 200 years for both vectors under 2 RCPs
**2. Scope**	Global	Europe & its selected 10 cities
**3. Focus**	1. Temperature and DTR on global DEP for *Ae. Aegypt*i.<br>2. Changes of global DEP from past to present and future under RCP8.5	1. Seasonality of European DEP for *Ae. aegypti* & *Ae. albopictus*<br>2. Climate change mitigation on European dengue transmission intensity and duration
**5. Vector**	*Ae. aegypti*	*Ae. aegypti & Aedes albopictus*
**6. Time scale**	**Calculation of *rVc* for *Ae. aegypti***	**Calculation of *VC* for *Ae. aegypti* & *Ae. albopictus***
**6.1 total time span & resolution**	200 years with 3 snapshots of 3 decades of each	Every decade over 200 years
**6.2 Seasonal maps**	The warmest 3 month average	Four seasons of the year
**6.3 Seasonality**	None	Every month of the year
**6.4 data input**	Monthly temperature	Daily and monthly

References

1 Jones, P. D. & Harris, I., University of East Anglia Climatic Research Unit; NCAS British Atmospheric Data Centre, 24th September 2014. (2014) CRU TS3.22: Climatic Research Unit (CRU) Time-Series (TS) Version 3.22 of High Resolution Gridded Data of Month-by-month Variation in Climate (Jan. 1901- Dec. 2013). [Accessed: 2014-10-02], available from: <http://dx.doi.org/10.5285/18BE23F8-D252-482D-8AF9-5D6A2D40990C >

2 Met Office: Met Office Integrated Data Archive System (MIDAS) Land and Marine Surface Stations Data (1853-current). NCAS British Atmospheric Data Centre (2012). [Access data: 2012-11-27], available from: <http://catalogue.ceda.ac.uk/uuid/220a65615218d5c9cc9e4785a3234bd0>

3 Jones, P. D. & Harris, I., University of East Anglia Climatic Research Unit; CRU TS3.10: Climatic Research Unit (CRU) Time-Series (TS) Version 3.10 of High Resolution Gridded Data of Month-by-month Variation in Climate (Jan. 1901 - Dec. 2009) [Accessed: 2012-09-27], available from: <http://catalogue.ceda.ac.uk/uuid/ac3e6be017970639a9278e64d3fd5508> (2013).

4 Massad, E. & Coutinho, F. A. B. Vectorial capacity, basic reproduction number, force of infection and all that: formal notation to complete and adjust their classical concepts and equations. *Mem. Inst. Oswaldo Cruz* **107**, 564-567 (2012).

5 Garrett-Jones, C. Prognosis for Interruption of Malaria Transmission through Assessment of the Mosquito's Vectorial Capacity. *Nature* **204**, 1173-1175 (1964).

6 Liu-Helmersson, J., Stenlund, H., Wilder-Smith, A. & Rocklov, J. Vectorial Capacity of Aedes aegypti: Effects of Temperature and Implications for Global Dengue Epidemic Potential. *PLoS One* **9**, e89783, doi:10.1371/journal.pone.0089783 (2014).

7 Brady, O. J. *et al.* Global temperature constraints on Aedes aegypti and Ae. albopictus persistence and competence for dengue virus transmission. *Parasit Vectors* **7**, 338 (2014).

8 European Centre for Disease Prevention and Control (ECDC). Mosquito maps. Aedes aegypti - Current known distribution - January 2016 & Aedes albopictus - Current known distribution - January 2016 (ECDC, Stockholm, 2016). [Accessed: 2016-02-17], available from<http://www.ecdc.europa.eu/en/healthtopics/vectors /vector-maps/Pages/VBORNET_maps.aspx> (2015).

9 Bhatt, S. *et al.* The global distribution and burden of dengue. *Nature* **12060**, doi:10.1038 (2013).

10 Medlock, J. M. *et al.* A review of the invasive mosquitoes in Europe: ecology, public health risks, and control options. *Vector-borne and zoonotic diseases* **12**, 435-447 (2012).

11 Whitehorn, J. *et al.* Comparative Susceptibility of Aedes albopictus and Aedes aegypti to Dengue Virus Infection After Feeding on Blood of Viremic Humans: Implications for Public Health. *J. Infect. Dis.*, doi:10.1093/infdis/jiv173 (2015).

12 Lambrechts, L., Scott, T. W. & Gubler, D. J. Consequences of the Expanding Global Distribution of Aedes albopictus for Dengue Virus Transmission. *PLoS Negl. Trop. Dis.* **4**, e646, doi:10.1371/journal.pntd.0000646 (2010).

13 Delatte, H. *et al.* Blood-feeding behavior of Aedes albopictus, a vector of Chikungunya on La Réunion. *Vector-Borne and Zoonotic Diseases* **10**, 249-258 (2010).

14 Delatte, H., Gimonneau, G., Triboire, A. & Fontenille, D. Influence of temperature on immature development, survival, longevity, fecundity, and gonotrophic cycles of Aedes albopictus, vector of chikungunya and dengue in the Indian Ocean. *J. Med. Entomol.* **46**, 33-41 (2009).

15 Lambrechts, L. *et al.* Impact of daily temperature fluctuations on dengue virus transmission by Aedes aegypti. *Proc. Natl. Acad. Sci. U. S. A.* **108**, 7460-7465, doi:10.1073/pnas.1101377108 (2011).

16 Scott, T. W. *et al. Longitudinal Studies of Aedes aegypti (Diptera: Culicidae) in Thailand and Puerto Rico: Population Dynamics*. Vol. 37 (2000).

17 Yang, H. M., Macoris, M. L., Galvani, K. C., Andrighetti, M. T. & Wanderley, D. M. Assessing the effects of temperature on the population of Aedes aegypti, the vector of dengue. *Epidemiol. Infect.* **137**, 1188-1202, doi:10.1017/S0950268809002040 (2009).

18 Macdonald, G. The analysis of equilibrium in malaria. *Tropical Disease Bulletin* **49**, 813-828 (1952).

19 Anderson, R. & May, R. *Infectious Diseases of Humans: Dynamics and Control*. (Oxford University Press, 1991).

20 Nishiura, H. & Halstead, S. B. Natural history of dengue virus (DENV)-1 and DENV-4 infections: reanalysis of classic studies. *J. Infect. Dis.* **195**, 1007-1013, doi:10.1086/511825 (2007).

21 Siler, J. F., Hall, M. W. & Kitchens, A. Dengue: Its History, Epidemiology, Mechanism of Transmission, Etiology, Clinical Manifestations, Immunity and Prevention. *Philippine Bur. Sci.* (1926).

22 Chan, M. & Johansson, M. A. The incubation periods of dengue viruses. (2012).

23 World Health Organization (WHO). *Dengue and severe dengue – Fact sheet N°117.* [Accessed: 2012-12-03], available from: <http://www.who.int/mediacentre/factsheets/fs117/en/# > (2012).
24 Brunette, G. W. *CDC Health Information for International Travel 2012: The Yellow Book: The Yellow Book*. (Oxford University Press, USA, 2011).
25 Centers for Disease Control and Prevention (CDC). *Mosquito-borne Transmission - Infectivity Period (about 7 days)*. [Accessed: 2015-12-01], available from: <http://www.cdc.gov/dengue/training/cme/ccm/page45915.html> (2015).
26 Quam, M. B., Sessions, O., Kamaraj, U. S., Rocklov, J. & Wilder-Smith, A. Dissecting Japan's Dengue Outbreak in 2014. *Am. J. Trop. Med. Hyg., 00(0), 2015, pp. 000–000* **In press**, doi:doi:10.4269/ajtmh.15-0468 (2015).
27 Amaku, M. *et al.* A Comparative Analysis of the Relative Efficacy of Vector-Control Strategies Against Dengue Fever. *Bull. Math. Biol.* **76**, 697-717, doi:10.1007/s11538-014-9939-5 (2014).
28 Pericchi, L. R. & Walley, P. Robust Bayesian credible intervals and prior ignorance. *International Statistical Review/Revue Internationale de Statistique*, 1-23 (1991).
29 Weyant, J. *et al.* in *IPCC* Vol. 2015 (IPCC Secretariat, Geneva, Switzerland, 2009).
30 Micieli, M. V. & Campos, R. E. Oviposition activity and seasonal pattern of a population of Aedes (Stegomyia) aegypti (L.) (Diptera: Culicidae) in subtropical Argentina. *Mem. Inst. Oswaldo Cruz* **98**, 659-663 (2003).
31 Rogers, D. J. Dengue: recent past and future threats. *Philosophical Transactions of the Royal Society of London B: Biological Sciences* **370**, 20130562 (2015).
32 Bouzid, M., Colón-González, F. J., Lung, T., Lake, I. R. & Hunter, P. R. Climate change and the emergence of vector-borne diseases in Europe: case study of dengue fever. *BMC Public Health* **14**, 781, doi:10.1186/1471-2458-14-781 (2014).
33 Naish, S. *et al.* Climate change and dengue: a critical and systematic review of quantitative modelling approaches. *BMC Infect. Dis.* **14**, 167-167, doi:10.1186/1471-2334-14-167 (2014).
34 Rogers, D. J., Suk, J. E. & Semenza, J. C. Using global maps to predict the risk of dengue in Europe. *Acta Trop.* **129**, 1-14 (2014).
35 Louis, V. R. *et al.* Modeling tools for dengue risk mapping-a systematic review. *International journal of health geographics* **13**, 1 (2014).
